# 3D Printed Flexible Piezoelectric Sensors for Integrated Hybrid Electronics

**DOI:** 10.1002/smll.202511146

**Published:** 2025-12-31

**Authors:** Daniel Wai Hou Ng, Hassene Ben Atitallah, Ghazaleh Haghiashtiani, Jinsheng Fan, Hyunjun Kim, Guebum Han, Riyan Mendonsa, Razman Zambri, Michael C. McAlpine

**Affiliations:** ^1^ Department of Mechanical Engineering University of Minnesota Minneapolis MN USA; ^2^ Seagate Technology LLC Bloomington MN USA

**Keywords:** 3d printing, human‐machine interfaces, light‐emitting diodes, piezoelectric polymers, wearable electronics

## Abstract

The ability to 3D print high performance smart materials and multifunctional devices, all seamlessly integrated via a common manufacturing platform, can yield advances in soft robotics, wearable electronics, and human‐machine interfaces. One of the most important smart materials in this context is piezoelectrics, due to their dual capabilities in sensing and actuating, which are critical for creating intelligent, responsive systems. In this study, we develop a direct‐ink‐writing (DIW) 3D printing approach for creating flexible and wearable piezoelectric devices using solution‐processed poly(vinylidene fluoride‐co‐trifluoroethylene) (PVDF‐TrFE) as the functional ink. Ferroelectric and actuation‐based characterizations are conducted to guide systematic optimization of the electrical poling conditions, yielding high performance 3D printed PVDF‐TrFE transducers with *d_31_* coefficient of 12.70 ± 0.71 pC·N^−1^. Three proof‐of‐concept smart devices were then fabricated: (1) a touch‐based wearable human‐machine interface for interactive gaming, (2) a tactile‐sensing “electronic skin,” and (3) a multifunctional hybrid electronic system combining piezoelectric sensors and quantum dot light‐emitting diodes, all fully 3D printed. This work comprehensively demonstrates the ability for 3D printing to generate high performance materials and devices, the use of 3D printing for wearable piezoelectric sensor fabrication, and the versatility of 3D printing for the seamless multifunctional integration of hybrid electronic systems.

## Graphical abstract

A comprehensive 3D printing strategy can fabricate high performance piezoelectric transducers on various substrates for wearable and flexible electronics applications. Demonstrations include human‐machine interfaces, tactile electronic skin, and a multifunctional system integrating piezoelectric sensors with quantum dot light‐emitting diodes. This versatile, multi‐material approach enables seamless integration of device classes, paving the way for fully 3D printed next‐generation multifunctional electronic systems.

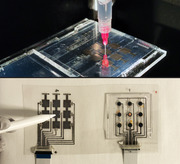

## 1 Introduction

Next‐generation electronics increasingly demand multifunctionality within a single integrated platform to enable seamless integration of diverse capabilities, such as sensing, energy harvesting, flexible displays, wireless communication, photonic and electronic computation, and energy storage [1]. Yet, achieving this integration remains challenging due to the disparate and often incompatible material properties and fabrication processes required for individual functional components [2]. For example, organic piezoelectric transducers are typically produced via electrospinning or spin‐coating to generate highly oriented polymer structures [3], while optoelectronic devices such as light‐emitting diodes (LEDs) and organic photodetectors (OPDs) depend on cleanroom‐based methods such as evaporation, spin‐coating, sputtering, photolithography, and chemical vapor deposition (CVD) [4]. These divergent fabrication requirements hinder multifunctional system integration, either due to material incompatibilities (e.g., lattice constant mismatches) or because the performance of individual devices may be compromised during the co‐fabrication of others [5]. A universal manufacturing approach that transcends these gaps is demanded [6].

Direct‐ink‐writing (DIW) 3D printing has recently emerged as a potential candidate to solve this challenge owing to several unique characteristics: (1) a compatibility with a wide range of functional materials [7], (2) the ability to deposit multiple materials in a single workflow [8], as well as to print on virtually any substrate, including non‐planar surfaces, and (3) an additive, localized process that selectively builds only on specified substrate areas, avoiding damage to other devices [9]. Indeed, DIW has shown applicability in fabricating diverse functional electronics, including quantum dot LEDs (QLEDs) [10], organic LEDs (OLEDs) [11], pressure sensors [12], soft actuators [13], temperature sensors [14], wireless power transmitters [15], and even bio‐integrated electronics [16]. Beyond its inherent versatility, DIW has achieved performance metrics comparable to traditional microfabrication processes. For example, Park et al. fabricated flexible OPDs via DIW under ambient conditions with achievable external quantum efficiencies (EQE) above 20%, on par with cleanroom‐fabricated devices [17]. More recently, Ghafari et al. reported DIW‐fabricated organic electrochemical transistors (OECTs) with a transconductance of 7.7 mS and a product of charge carrier mobility and volumetric capacitance of 141 F·cm^−1^·V^−1^·s^−1^, comparable to spin‐coated devices (5.6 mS, 106 F·cm^−1^·V^−1^·s^−1^) [18]. These advances are impressive considering that the bulk of the 3D printing research community has focused on bioprinting rather than functional materials printing, foreshadowing the potential for 3D printing to emerge as an elite unified fabrication strategy for multifunctional and high performance electronic systems.

Amidst these advances, interest has surged in using 3D printing to fabricate high performance piezoelectric transducers, particularly those based on poly(vinylidene fluoride) (PVDF), due to its inherent flexibility and utility in sensing, actuation, and energy harvesting [19]. However, research in this area remains at a nascent stage, and a disconnect persists between material‐level studies and practical engineering efforts to develop devices. Traditional techniques, including electrospinning, spin‐coating, and solution casting, have provided key insights into PVDF optimization [20, 21]. They often require the piezoelectric layer to be fabricated on a separate carrier substrate and later transferred to the target surface, which introduces an additional fabrication step and can lead to defects and reduced device sensitivities. As a result, these studies have focused more on fundamental material characterization and optimization than practical device development. In contrast, emerging 3D printing techniques, including fused deposition modeling (FDM) and DIW, have demonstrated functioning piezoelectric devices, yet they often lack comprehensive material characterization to guide process optimization [22, 23, 24, 25]. For example, Fan et al. applied a low poling electric field (14 MV·m^−1^) to their FDM‐printed PVDF pressure sensors, requiring a prolonged 4 h poling duration to induce measurable piezoelectric effects [24]. Similarly, Yan et al. carried out an extended 8 h poling at 10 MV·m^−1^ for their DIW‐printed PVDF‐TrFE energy harvester, yielding a piezoelectric performance of *d_33_* = −16 pC·N^−1^, requiring the incorporation of inorganic fillers to enhance the performance (*d_33_* = −30 pC·N^−1^) [23]. Other 3D printing studies on PVDF‐based transducers have omitted electrical poling and relied entirely on filler composites [26, 27]. Concerns persist regarding the repeatability of such approaches and whether the observed signals originate from triboelectric or electrostatic effects rather than true piezoelectricity [28]. More critically, these studies have remained limited to standalone piezoelectric devices. To date, no 3D printing workflow has demonstrated the co‐fabrication of PVDF‐based transducers alongside other active elements on a single flexible substrate, a task that is equally challenging for conventional methods.

**FIGURE 1:**
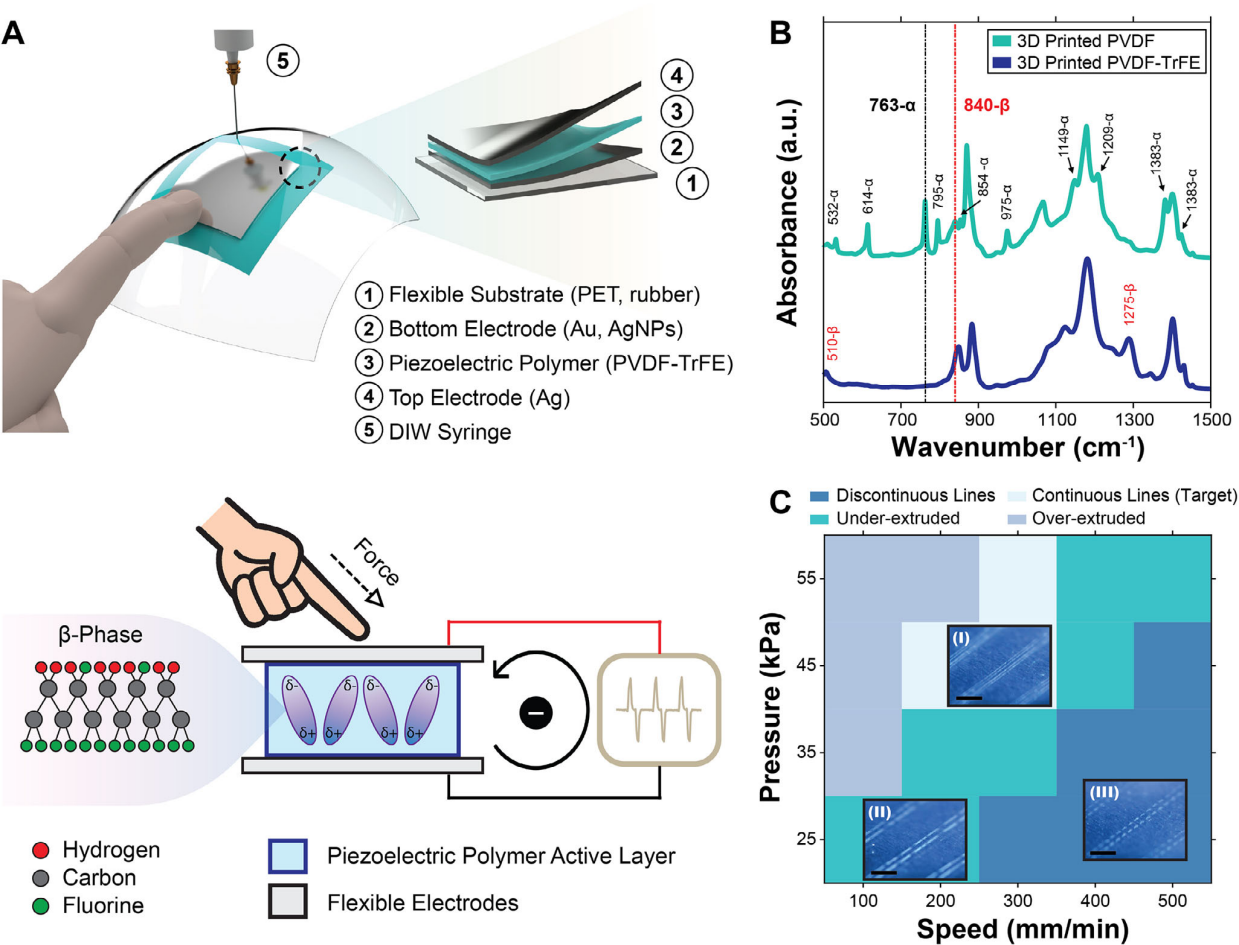
3D printing of piezoelectric polymers. (A) Schematic illustration showing (top) the DIW process and the layer structure of the piezoelectric polymer sensor on a flexible substrate, and (bottom) the generation of piezoelectric‐induced current in response to applied mechanical force. The molecular diagram depicts the structure of β‐phase PVDF‐TrFE. (B) FT‐IR spectra of 3D printed PVDF and PVDF‐TrFE (a.u. indicates arbitrary units). (C) Evaluation of PVDF‐TrFE print lines under different printing speeds and pressures using a 25‐gauge nozzle. Inset (I) shows continuous print lines with the desired width and height; inset (II) shows under‐extruded print lines with mild defects; inset (III) shows discontinuous print lines (scale bars = 2.5 mm).

To address these critical gaps, we developed a comprehensively optimized 3D printing approach to fabricate high performance piezoelectric tactile sensors based on poly(vinylidene fluoride‐co‐trifluoroethylene) (PVDF‐TrFE) (Figure 1A). Our work investigated the ferroelectric properties of the PVDF‐TrFE active layer, which is critical to extract key parameters such as the coercive electric field, thereby guiding the optimization of electrical poling treatments. Moreover, we quantitatively calculated the piezoelectric coefficients (*d_31_*) of our 3D printed PVDF‐TrFE transducers using both inverse and direct piezoelectric responses. This cross‐validation not only confirmed that the observed electrical responses originated from true piezoelectricity, but also established robust metrics for comparison against literature values. Furthermore, to demonstrate the practical utility of this optimized 3D printing process, we developed two wearable sensor prototypes. First, a nitrile glove embedded with two PVDF‐TrFE sensors enabled intuitive and accurate (>95%) real‐time control of a 2D character in a virtual environment. Second, an “electronic skin” composed of five tactile sensing elements that illuminated corresponding connected LEDs upon mechanical touch, showcasing the potential for high precision human‐machine interactions and real‐time tactile monitoring. Finally, to showcase the versatility of the DIW 3D printing workflow, we fabricated a fully 3D printed hybrid electronic system, wherein a 3 × 3 PVDF‐TrFE sensor array was seamlessly printed alongside a 3 × 3 quantum dot LED (QLED) matrix onto a single polyethylene terephthalate (PET) substrate. This proof‐of‐concept multifunctional device validates the ability of DIW to produce high performance piezoelectric components, as well as its realization as a universal platform for co‐fabricating diverse active materials and functional devices for next‐generation flexible and wearable electronics.

## 2 Results and Discussion

### 2.1 Electroactive Crystalline Content

3D printed pure PVDF often exhibits low piezoelectric performance due to the predominance of the thermodynamically stable α‐phase formed during melt‐solidification [29]. This phase features antiparallel dipole alignment, which neutralizes net polarization and reduces piezoelectric activity [30]. By contrast, the β‐phase, which is primarily responsible for the piezoelectric characteristics, consists of two all‐trans polymer chains with dipoles aligned in the same direction. In conventional methods such as spin‐coating and electrospinning, this highly polar β‐phase is typically induced via electrical poling combined with mechanical stretching and thermal treatment [31]. However, applying mechanical stretching is impractical for 3D printed devices designed for conformal or in situ applications. To overcome this, several strategies have been proposed to enhance β‐phase content without relying on stretching. For instance, studies have suggested that carefully controlled shear and elongational forces imparted by the nozzle during the 3D printing process can help align polymer chains into the all‐trans conformation [32], and more recent work has shown that incorporating architected metamaterials can further promote self‐poling in PVDF [33]. Alternatively, certain PVDF copolymers, such as poly(vinylidene fluoride‐co‐trifluoroethylene) (PVDF‐TrFE), naturally exhibit a higher β‐phase content as the incorporation of TrFE units facilitates the all‐trans conformation of PVDF chains, thereby promoting spontaneous β‐phase formation even without external poling or stretching [30].

In this work, PVDF‐TrFE was selected over its parent polymer, PVDF, as the piezoelectric active material, capitalizing on its naturally high β‐phase crystallinity. This eliminates the need for mechanical stretching and thermal treatment during electrical poling, rendering it well‐suited for DIW 3D printing. This increased β‐phase crystalline content is validated through Fourier‐transform infrared (FT‐IR) spectroscopy measurements, comparing 3D printed PVDF and PVDF‐TrFE films (Figure 1B). The peak at 763 cm^−1^ for PVDF indicates the electromechanically inactive α‐phase, whereas the peak near 840 cm^−1^ in PVDF‐TrFE confirms a high β‐phase crystalline content. To provide a quantitative comparison, the β‐phase crystalline content of these materials is calculated using Equation (1) [34].

(1) $$F\left(\beta \right)=\frac{A_{\beta }}{\left(\frac{K_{\beta }}{K_{\alpha }}\right)A_{\alpha }+A_{\beta }}$$

Here, F(β) is the fraction of β‐phase crystalline, A_α_ is the measured absorption band at 763 cm^−1^, A_β_ is the measured absorption band at 840 cm^−1^, K_α_ is the absorption coefficient at 763 cm^−1^, and K_β_ is the absorption coefficient at 840 cm^−1^.

Notably, recent studies suggest that the F(β) calculated using Equation (1) more accurately represents the electroactive crystalline fraction, which includes β‐ and γ‐phases (both are piezoelectric) rather than pure β‐phase alone [27, 35]. Nevertheless, it remains a widely adopted and valuable indicator of piezoelectric performance in the literature. We adopted this metric to facilitate comparison with existing studies. Based on our FT‐IR measurements, the 3D printed PVDF samples exhibited a β‐phase crystalline fraction of 35.65 ± 0.91% (*n* = 5), whereas the 3D printed PVDF‐TrFE samples possessed a β‐phase crystalline fraction of 87.03 ± 0.85% (*n* = 5). This greater than twofold increase in electroactive crystalline content confirms the suitability of PVDF‐TrFE as a superior alternative to pure PVDF for DIW‐based 3D printing. Table 1 presents a comparison of electroactive crystalline content for PVDF‐TrFE fabricated via various methods.

**TABLE 1:** Comparison of electroactive crystalline content, coercive field (E_c_), and remnant polarization (P_r_) for different fabrication methods of PVDF‐TrFE transducers.

Fabrication Method	F(*β*) (%)	*E_c_* (MV·m^−1^)	*P_r_* (µC·cm^−2^)
Screen printing [36]	—	48	7.4
Doctor blade coating [35, 37]	96.8 [35]	55 [37]	∼2 [37]
Spin‐coating [38, 39]	73.6 [39]	53 [38]	4.7 [38]
Electrospinning [40, 41]	79.0 [41]	∼35 [40]	4.3 [40]
FDM 3D printing [42]	—	65	> 8
DIW [23]	∼82	68.2	2.61
DIW (This work)	87.03	58.33	5.97

### 2.2 Optimization of 3D Printing Parameters

We then investigated the influence of the printing parameters on the fidelity and geometry of printed piezoelectric features. The 3D printing technique in this work utilized a 3D gantry system to control the traveling speed of the print head, while a pneumatic pressure regulator provided the necessary pressure to extrude the piezoelectric functional inks. These printing parameters play critical roles in determining the line thickness (or layer height) and the line width of the printed features. The thickness directly influences the required poling voltage for the piezoelectric film, whereas the line width reflects the degree of ink flow.

To investigate these influences, PVDF‐TrFE ink (70/30 molar ratio, 15 wt.% concentration in a mixed N,N‐dimethylformamide:acetone solvent) was printed using a 25‐gauge stainless‐steel nozzle under varying printing speeds and extrusion pressures, and the thicknesses and widths of the resulting features were then measured. The ink exhibited shear‐thinning behavior, which made it suitable for DIW printing (Figure S1). Furthermore, as anticipated, both thickness and width increased with higher printing pressure and decreased with faster printing speeds (Figure S2). The printed features exhibited a thickness range of 2–12 µm and a width range of 150–815 µm. Based on these findings and visual analysis, a qualitative evaluation was conducted to identify optimal printing conditions, targeting a thickness between 5 and 10 µm and a width between 400 and 550 µm, which were found to be well‐suited for downstream processing and device integration. Figure 1C summarizes the evaluation results, with insets showing representative examples of printed PVDF‐TrFE lines that were discontinuous, under‐extruded, or showed desired continuity. It should be noted that under‐extruded print lines were defined as those with thicknesses below 5 µm or widths below 400 µm, which may occasionally yield minor defects. Nonetheless, such features might still be appropriate when a thinner film is desired, and for situations when the yield rate is not the highest priority. In contrast, over‐extruded print lines were defined as those exceeding 10 µm line thickness, as they would require higher voltages during the poling process. As such, the optimal printing condition was determined to be a printing pressure of 45 kPa and a printing speed of 200 mm min^−1^, which consistently produced well‐defined, continuous features with 508.50 ± 9.43 µm widths and 7.23 ± 0.16 µm layer heights (*n* = 5).

### 2.3 Characterization and Optimization of 3D Printed Piezoelectric Polymers

**FIGURE 2:**
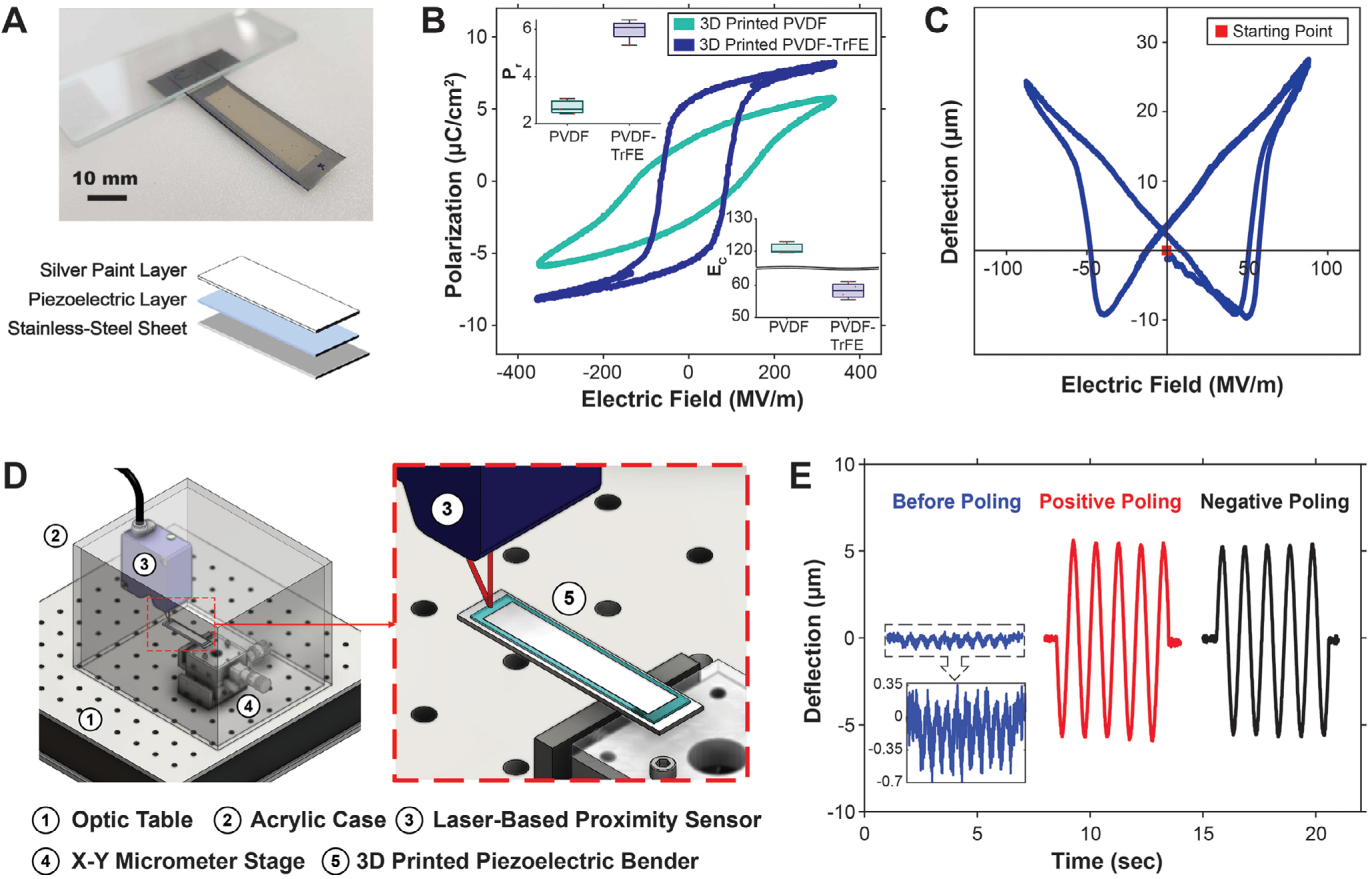
Characterization of 3D printed piezoelectric polymers. (A) Image and layer structure of the piezoelectric benders. Dimensions: stainless steel substrate (12 mm × 45 mm × 0.1 mm), 3D printed piezoelectric layer (10 mm × 35 mm), and 3D printed silver paint top electrode layer (8 mm × 30 mm). (B) Hysteretic P‐E curves obtained from 3D printed PVDF and PVDF‐TrFE benders. Insets compare the coercive field (E_C_, unit is MV·m^−1^) and remnant polarization (P_r_, unit is µC·cm^−2^) between 3D printed PVDF (n = 3) and PVDF‐TrFE (n = 4). (C) Butterfly plot of a 3D printed PVDF‐TrFE bender. (D) Schematic showing the testing setup used to measure displacement/deflection at a target point on the 3D printed PVDF‐TrFE benders (installed in a cantilever configuration). (E) Deflection of the same PVDF‐TrFE bender under identical driving conditions (sinusoidal at 1 Hz, ± 200 V, 5 cycles) subjected to (left) no poling, (middle) poling at +130 MV·m^−1^ for 1 s, and (right) poling at −130 MV·m^−1^ for 1 s. The poling treatments were applied in the stated order.

Using the optimized printing parameters, a simple single‐layer (also known as unimorph) piezoelectric bender convenient for testing and characterization was designed and 3D printed (Figure 2A). Two variants of piezoelectric benders were fabricated: one with a 3D printed PVDF active layer and another with a 3D printed PVDF‐TrFE active layer. In both cases, the active piezoelectric layer was sandwiched between a thin stainless‐steel sheet (cathode) and a layer of 3D printed silver paste (anode). We measured the hysteretic polarization‐electric field (*P‐E*) loops from both piezoelectric benders (Figure 2B) using a custom‐built Sawyer‐Tower circuit (Figure S3) to evaluate their ferroelectric properties. This allowed the extraction of key ferroelectric parameters such as the coercive electric field and remnant polarization, both of which are critical for understanding and optimizing the piezoelectric performance. The coercive field (*E_C_*) quantifies the minimum electric field required to reorient the dipoles within the electroactive β‐phase, directly influencing the poling voltage magnitude. Meanwhile, the remnant polarization (*P_r_*) reflects the retention of dipole orientation after the electrical poling treatment, such that higher values typically correlate with enhanced piezoelectric responses.

It was found that 3D printed PVDF (*n* = 3) possessed a coercive field of 120.78 ± 1.50 MV·m^−1^ and remnant polarization of 2.70 ± 0.27 µC·cm^−2^, whereas 3D printed PVDF‐TrFE (*n* = 4) possessed a coercive field of 58.33 ± 2.14 MV·m^−1^ and remnant polarization of 5.97 ± 0.40 µC·cm^−2^. The lower coercive field of PVDF‐TrFE indicated that it required a lower poling electric field, and consequently a lower poling voltage, to achieve effective crystalline alignment compared to pure PVDF. Additionally, its higher remnant polarization is advantageous for achieving high piezoelectric performance in various applications. Once again, these findings underscore the suitability of PVDF‐TrFE as a superior material for the DIW process. For reference, Table 1 summarizes key ferroelectric parameters of PVDF‐TrFE produced by different methods, including those from this study.

**FIGURE 3:**
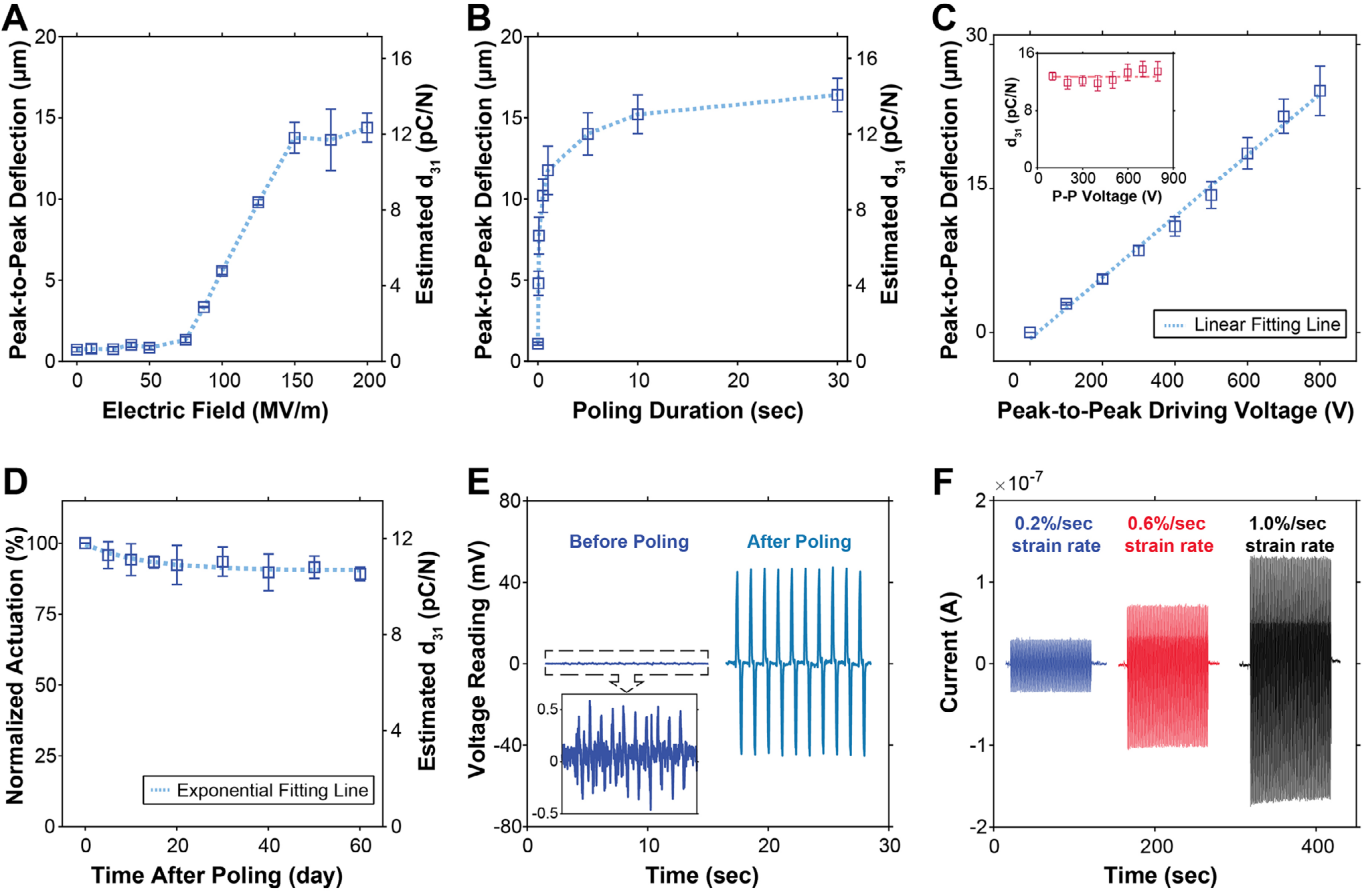
Optimization of electrical poling conditions and characterization of 3D printed piezoelectric sensors. (A) Actuation of 3D printed PVDF‐TrFE benders (n = 3) as a function of poling electric field (fixed duration of 5 s). (B) Actuation of 3D printed PVDF‐TrFE benders (n = 3) as a function of poling duration (poling electric field of +150 MV·m^−1^). (C) Actuation of 3D printed PVDF‐TrFE benders (n = 4) treated with optimized poling conditions (+150 MV·m^−1^, duration 5 s) as a function of driving voltage (sinusoidal at 1 Hz, 5 cycles). R^2^ = 0.993. The inset shows the estimated d_31_ piezoelectric coefficient as a function of driving voltage (n = 4). (D) Aging characterization of the actuation performance of 3D printed PVDF‐TrFE benders (n = 4) as a function of time elapsed after the poling treatment. R^2^ = 0.885. (E) Direct piezoelectric response from the same 3D printed PVDF‐TrFE bender under a controlled input deformation (±1 mm, 500 mm·min^−1^, 10 cycles) before and after poling treatment (+ 150 MV·m^−1^, 5 s). (F) Direct piezoelectric responses from a standalone 3D printed PVDF‐TrFE sensor (poled at + 150 MV·m^−1^, 5 s) under 100 cycles of sinusoidal strains of different strain rates (0.2%/sec, 0.6%/sec, and 1.0%/sec). Cyclic strains were supplied using an RSA‐G2 dynamic mechanical analyzer (TA Instruments).

Building on the ferroelectric insights, we next characterized and optimized the piezoelectric performance via electrical poling. While this work focuses on 3D printed PVDF‐TrFE as sensors (direct piezoelectric effect), most of the fundamental characterizations were conducted through actuation measurements (inverse piezoelectric effect). This approach was chosen because multiple physical phenomena can contribute to generating an electrical signal from physical touch, including the triboelectric effect [43], electrostatic induction [44], flexoelectricity [45], and contact electrification [46]. These effects can introduce significant “noise,” comparable to or even exceeding the magnitude of the actual piezoelectric signal, making it challenging to isolate the true piezoelectric response [28]. In contrast, the inverse piezoelectric effect has distinct characteristics compared to other voltage‐driven actuation mechanisms, such as electrostatic (capacitive) actuation [47], electrostrictive actuation [48], dielectric elastomer actuation (DEA) [13], and ionic electroactive polymer (IEAP) actuation [49], making it easier to extract the true piezoelectric performance while minimizing interference from unwanted effects. For instance, a key indicator of piezoelectric actuation is the characteristic “butterfly curve” (Figure 2C), a unique actuation behavior commonly observed in both inorganic and organic piezoelectric benders [50, 51]. This butterfly curve was obtained from a 3D printed PVDF‐TrFE bender by applying a sinusoidal electric field with peak values substantially exceeding the coercive field of the material and measuring the tip displacement of the bender using a high‐resolution laser‐based proximity sensor, as illustrated in Figure 2D. This setup was also employed for subsequent experiments shown in Figures 2E and 3A–D.

Another defining feature of piezoelectric actuation is its polarity dependence. Unlike electrostatic (in parallel plate configuration) and electrostrictive actuators, which induce motion in only one direction regardless of voltage polarity, piezoelectric actuators exhibit bidirectional actuation. While DEA and IEAP also exhibit bidirectional actuation, piezoelectric actuation is “programmable,” as its actuation direction is strongly dependent on the poling treatment. For instance, Figure 2E presents three actuations of the same PVDF‐TrFE piezoelectric bender under identical driving voltage signals (sinusoidal at 1 Hz, ±200 V, 5 cycles) while subjected to different poling processes. The first curve, corresponding to the as‐fabricated PVDF‐TrFE bender prior to any poling treatment, exhibited minimal actuation in a single direction. Additionally, the presence of 10 actuation “peaks,” corresponding to the 10 extrema of the driving voltage (5 positive and 5 negative), further confirmed that the unpoled sample was governed by unidirectional actuation mechanisms, as discussed previously. The second curve, obtained after applying a positive poling treatment, demonstrated a substantially larger actuation magnitude and a bidirectional response that closely followed the sinusoidal driving voltage (see Figure S4 for synchronized voltage and displacement data). This alignment confirms that the actuation was primarily due to the inverse piezoelectric effect. The third curve, on the other hand, underwent a negative poling treatment and hence exhibited an opposite bidirectional actuation behavior compared to the second curve despite experiencing the same input voltage signal, further demonstrating the “programmability” of our 3D printed piezoelectric bender.

We then determined the optimal electrical poling parameters to achieve high performance 3D printed PVDF‐TrFE. To do so, a series of 3D printed PVDF‐TrFE benders (*n* = 3) were subjected to increasing poling electric fields while maintaining a fixed poling duration of 1 s. After poling, each bender was tested with an identical driving voltage signal (sinusoidal at 1 Hz, ±250 V, 5 cycles) to evaluate the actuation response. As the driving voltage was sinusoidal, the average peak‐to‐peak actuation was used as the metric for performance comparison. Figure 3A shows that the actuation remained minimal until the poling electric field reached a “knee point” at 75 MV·m^−1^, beyond which the actuation response increased sharply, followed by a saturating point at 150 MV·m^−1^, which we identified as the optimal poling electric field. Notably, this finding aligns with the results reported by Ducrot et al. for spin‐coated PVDF‐TrFE micro‐resonators, where poling became effective beyond 50 MV·m^−1^ and saturated near 100 MV·m^−1^ [20]. Notably, our knee point value of 75 MV·m^−1^ closely matches the previously measured coercive field of 3D printed PVDF‐TrFE (58.33 ± 2.14 MV·m^−1^, *n* = 4), which represents the minimum electric field required to align the electroactive crystalline structure. Moreover, upon close observation of the actuation behavior of the bender at a few critical poling electric fields (Figure S5), the actuations below the knee point were primarily unidirectional. Beyond the knee point, the bidirectionality progressively developed as the electric field increased. This emphasizes the importance of ferroelectric characterization in guiding electrical poling optimization.

Next, with a poling electric field of +150 MV·m^−1^, a new series of PVDF‐TrFE benders (*n* = 3) was subjected to increasing poling durations and underwent the same testing procedure as previously described. Results indicated that the piezoelectric actuation improved considerably even with short poling durations (Figure 3B). Specifically, a 5‐second poling duration achieved approximately 85% of the actuation amplitude obtained at 30 s, while the risk of dielectric breakdown became more pronounced beyond 5 s. Therefore, a 5‐second poling duration was selected as optimal for this work. Notably, when the *x*‐axis was plotted on a logarithmic scale, the piezoelectric response exhibited an approximately linear relationship with the logarithm of poling duration up to 30 s (Figure S6). Additionally, a new series of 3D printed PVDF‐TrFE benders (*n* = 4) was subjected to the optimal poling conditions (150 MV·m^−1^, 5 s), and the relationship between input voltage and actuation magnitude was measured (Figure 3C). The linear proportionality observed in the data further ruled out alternative actuation mechanisms such as electrostatic actuation [52], electrostrictive actuation [53], and DEA [54], all of which exhibit a quadratic dependence on the input voltage. An aging test was conducted on the same series of piezoelectric benders to assess their piezoelectric stability over time (Figure 3D). The results showed that the benders retained 89.13 ± 2.41% (*n* = 4) of their initial piezoelectric response 60 days post‐poling, demonstrating long‐term effectiveness of our proposed electrical poling strategy.

Notably, an estimation of the piezoelectric coefficient *d_31_* of the 3D printed benders, which is a crucial metric to compare piezoelectric performance, can be calculated through Equation (2) based on their actuation behavior (the detailed derivation of the equation is included in the Supporting Information, along with Figures S7–S10) [55].

(2) $$d_{31}\approx -\frac{1}{3}\left[\frac{Y_{b}{\left(t_{b}\right)}^{2}}{Y_{p}\left(1-v_{b}\right)\left[x_{1}\left(2x_{2}-x_{1}\right)\right]}\right]\left(\frac{w_{b}}{w_{P}}\right)\cdot \frac{\Delta z_{x_{2}}}{V_{in}}$$

where Y_b_ is the Young's modulus of the substrate (N m^−2^), *t_b_* is the thickness of the substrate (m), *v*_b_ is Poisson's ratio of the substrate, *x_1_*, *x_2_* are length of piezoelectric layer from edge and distance to measurement point from edge (m), respectively (see Figure S9), *w_b_* is the width of the substrate (m), *w_p_* is the width of the piezoelectric layer (m), Δz*_x2_* is the piezoelectric‐induced displacement at the measurement point (m), and V_in_ is the driving voltage (V).

The estimations of *d_31_* for the piezoelectric benders used in Figure 3A–D were calculated and included in the plots. The estimated *d_31_* value of 3D printed PVDF‐TrFE benders that underwent optimal poling conditions (Figure 3C) was 12.70 ± 0.71 pC·N^−1^ (*n* = 4). Notably, this short yet effective poling treatment produced piezoelectric performance comparable to PVDF‐TrFE transducers fabricated via conventional methods with electrical poling, as shown in Table 2.

**TABLE 2:** Comparison of piezoelectric coefficients for different fabrication methods and poling conditions of PVDF‐TrFE transducers.

Fabrication Method	Poling Conditions	Piezoelectric Coefficients (pC·N^−1^) or (pm·V^−1^)
Screen printing [36]	112.5 MV·m^−1^, duration not reported	*d_31_* = 7, *d_33_* = −23
Bar coating [56]	70 MV·m^−1^, 15 min	*d_31_* = 12, *d_33_* = −28
Spin‐coating [20]	100 MV·m^−1^, 5 min	*d_31_* = 11
Electrospinning [57]	100 MV·m^−1^, 30 min	*d_31_* = 15.73
DIW [23, 27]	i. No post‐poling [27] ii. 10 MV·m^−1^, 8 h [23]	i. *d_33_* = −1.9 [27] ii. *d_33_* ∼ −16 [23]
DIW (This work)	150 MV·m^−1^, 5 s	*d_31_* = 12.70

Following this, we sought to confirm that the previously identified optimal poling conditions effectively maximized the direct piezoelectric response (sensing) of PVDF‐TrFE devices. Additionally, we aimed to verify whether the piezoelectric coefficient *d_31_* calculated from the sensing performance aligns with the value obtained from actuation‐based measurements. To achieve this, a PVDF‐TrFE bender was prepared, and a 3D gantry system was programmed to impose a series of controlled deformations with fixed parameters (±1 mm displacement, 500 mm min^−1^ speed, 10 cycles) on the same bender, prior to and after the optimal poling treatment. The two electrodes of the bender were connected in parallel across a 10 MΩ resistor, converting the piezoelectric‐induced current into a voltage signal according to Ohm's law. This voltage signal was then recorded using a data acquisition unit (NI myDAQ). The results showed that the unpoled PVDF‐TrFE bender produced a weak electrical response in the range of ±0.5 mV, potentially attributable to residual piezoelectricity from slight preferred crystalline alignment in the as‐fabricated sample or other contact‐induced current effects, as previously discussed. However, after poling, this signal exhibited an approximately hundred‐fold increase (Figure 3E), confirming that the poling process significantly enhances the piezoelectric‐induced sensing capability, as it does for actuation.

For further validation, a standalone PVDF‐TrFE thin film, coated with 100 nm gold layers on both sides, was specifically designed, 3D printed, and poled for dynamic mechanical analysis (DMA). In this sample, thin gold layers replaced silver paint electrodes to minimize mechanical interference during the DMA testing. During the measurement, cyclic strains at 1 Hz were applied to the standalone PVDF‐TrFE thin film (Figure S7), and the resulting electrical signals were recorded via the NI myDAQ (Figure 3F). Simultaneously, the force applied to the sample was recorded using the DMA testing equipment itself. By utilizing the force and electrical signal data, the *d_31_* piezoelectric coefficient was computed using Equation (3) [58]. A detailed example calculation is provided in Supporting Information.

(3) $$D_{3}=d_{31}T_{1}+{\varepsilon_{33}}^{T}E_{3}$$

Here, *D_3_* is the electric charge displacement density in the thickness direction (C·m^−2^), *d_31_* is the piezoelectric coupling coefficients in strain‐charge form (C·N^−1^), *T_1_* is the input stress along the force direction (N·m^−2^), *ε^T^* is electric permittivity of the material (F·m^−1^), and *E_3_* is the applied electric field along the thickness direction (V·m^−1^).

Using the results from the DMA experiment, the estimated *d_31_* values for the standalone thin film subjected to 0.2% strain/sec, 0.6% strain/sec, and 1.0% strain/sec were 11.82 ± 1.42 pC·N^−1^ (*n* = 96), 10.95 ± 0.77 pC·N^−1^ (*n* = 96), and 10.70 ± 0.53 pC·N^−1^ (*n* = 96), respectively. These values are closely aligned with the previously determined *d_31_* value estimated from piezoelectric benders, further validating the consistency of the measurements. This strong agreement between sensing‐based and actuation‐based *d_31_* calculations enhances the credibility and reliability of the measured piezoelectric properties, further confirming the robustness of our experimental approach. Notably, both experiments were conducted at 1 Hz excitations (i.e., voltage for benders and strain for DMA), which falls within the quasi‐static regime and is therefore compatible with standard low‐frequency piezoelectric coefficient characterization methods.

Although this work did not focus on optimizing the 3D printed PVDF‐TrFE devices for energy harvesting applications (for example, no impedance matching was performed), their performance in this context can still be evaluated using the current signals recorded during DMA measurements, as analyzed using Equations (4) and (5).

(4) $$P_{avg}=\frac{I_{0}^{2}}{2}\cdot R$$

(5) $$E=\int_{t_{0}}^{t_{f}}I^{2}\left(t\right)\cdot Rdt\approx \sum_{i=1}^{n-1}I^{2}\left(t_{i}\right)\cdot R\cdot \Delta t$$

Here, *P_avg_* is the average output power from a sinusoidal current signal (W), *I_0_* is the peak current (A), *R* is the load resistance (Ω), and *E* is the total energy harvested over time *t* (J).

Using the current signal generated under ±0.5% sinusoidal strain at 1 Hz, and a load resistance of 10 MΩ, the device achieved: 1) an average power output of 0.11 µW, 2) an average power density of 26.86 nW cm^−2^ (or 12.79 µW cm^−3^), and 3) a total energy generation of 9.19 µJ over a 100‐second duration. When compared with previously reported pure polymeric PVDF‐based energy harvesters, the performance of our 3D printed device is within the same order of magnitude when operating under low‐frequency mechanical deformation, which are conditions relevant to wearable and biomechanical environments [59].

### 2.4 Wearable Tactile Sensing

**FIGURE 4:**
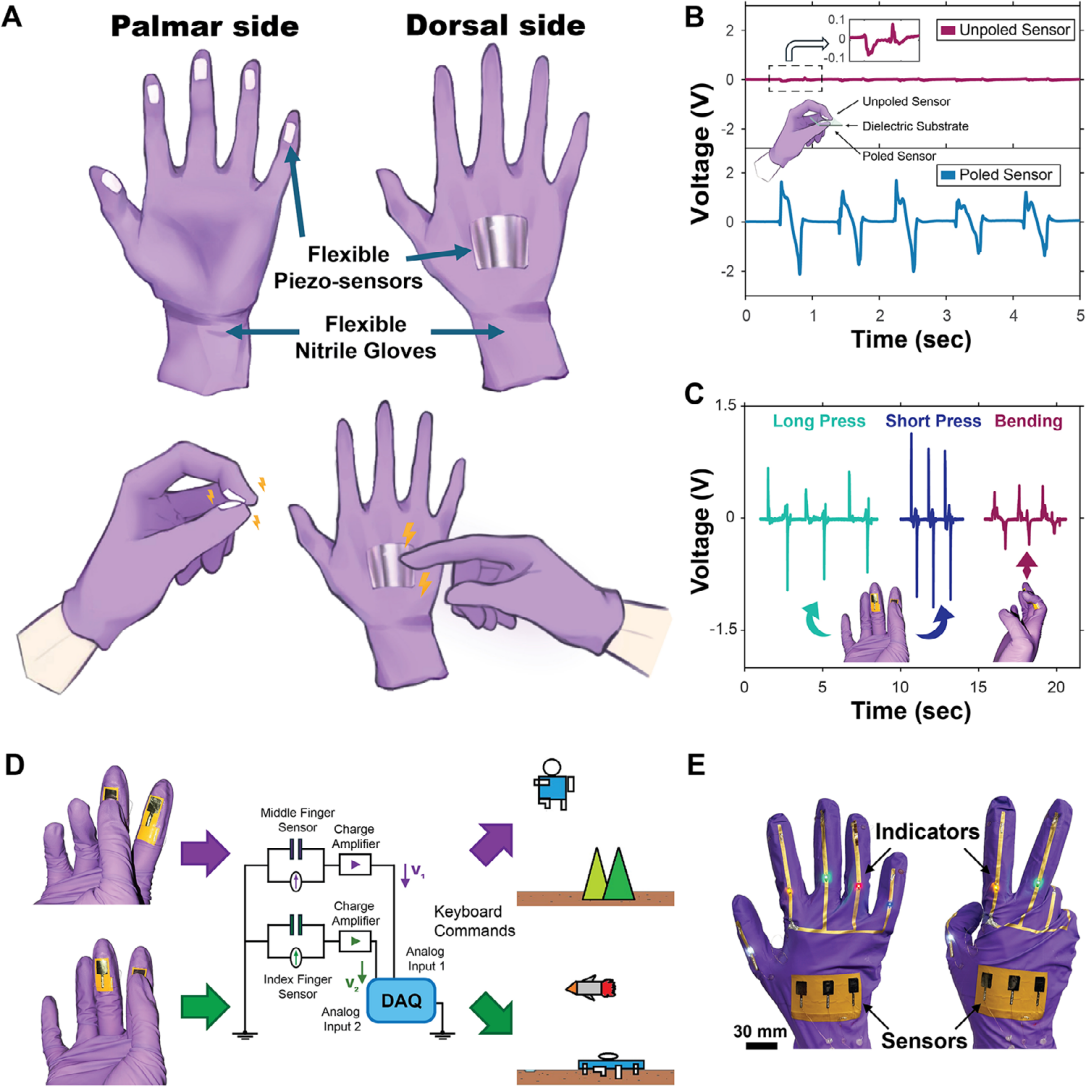
3D printed wearable tactile sensors. (A) Illustration of 3D printed flexible tactile sensors positioned at various locations of a nitrile rubber glove (piezo‐sensors indicate piezoelectric sensors). The bottom‐left schematic shows their use as active sensors enabling a human–machine interface, while the bottom‐right schematic demonstrates passive detection of external touches. (B) Comparison of electrical signals from passive (unpoled) and active (poled) sensors when pressed against a common dielectric substrate. (C) Electrical signal patterns generated by different finger motions. (D) Schematic of the circuit and logic of an interactive gaming system using 3D printed wearable sensors. (E) Images of 3D printed wearable tactile sensors integrated with commercial LED chips as touch indicators.

These systematically optimized 3D printed piezoelectric transducers could find potential in advancing wearable sensing technologies. We developed two proof‐of‐concept applications using the 3D printed PVDF‐TrFE sensors fabricated on nitrile rubber substrates (Figure 4A). The first application served as a human‐machine interface for sending active commands, showcasing the potential for integration into virtual reality (VR) systems. The second application utilized 3D printed PVDF‐TrFE as tactile sensor nodes to passively detect touches, and commercial LED chips were used to signal the presence of physical interactions with the sensors. We envision this lightweight and flexible sensor system can be scaled to thousands of nodes, making it a promising approach for next‐generation electronic skin.

Prior to the demonstrations, it is necessary to once again confirm that the electric signals generated by the sensors with nitrile rubber substrates primarily originated from the direct piezoelectric effect, for which two 3D printed PVDF‐TrFE devices were fabricated and tested: one as‐fabricated (unpoled) and one poled under optimal conditions. These sensors were squeezed against one another, with a dielectric material (a cleaned microscope glass slide) placed between them to prevent direct contact and to eliminate potential triboelectric signal interference (Figure 4B). According to Newton's Third Law of Motion, both sensors should experience equal force in opposing directions at the same rate. Each sensor was connected in parallel across a 100 MΩ resistor to convert piezoelectric‐induced current into voltage signals, and a NI myDAQ was employed to record the electrical signals from both sensors simultaneously. Figure 4B compares the output from the two PVDF‐TrFE sensors, showing that the poled sensor generated an electric signal nearly 20× stronger than that of the unpoled sample. A signal‐to‐noise ratio (SNR) analysis was also performed, and the unpoled and poled sensors exhibited SNR values of −12.21 and 18.17 dB, respectively (Supporting Information, along with Figures S11–S14). Notably, both sensors exhibited similar signal patterns despite the disparity in signal magnitude. This suggests that the weak signal observed in the unpoled sensor likely originated from the slight preferred crystalline orientation in the as‐fabricated film, which imparted a small degree of residual piezoelectricity. Interestingly, this spontaneous polarization appeared to be oriented opposite to that of the poled sensor, resulting in similar signal waveforms with reversed polarity.

Figure 4C presents the electric signals generated by a 3D printed sensor in response to three distinct finger motions, highlighting their potential for gesture recognition and next‐generation human‐machine interface systems. These flexible sensors could accurately differentiate between various finger movements, and their corresponding signal patterns can be processed by machine learning algorithms to enable real‐time gesture recognition. Once trained, the system could map these gestures to specific commands, allowing seamless interaction with digital environments. To illustrate this concept, we conducted a simple proof‐of‐concept demonstration, showcasing the integration of these sensors into an interactive control system. A nitrile glove embedded with two 3D printed, poled PVDF‐TrFE sensors was used to control a digital character in a 2D jumping game (Figure 4D). Moreover, Movie S1 showed that this flexible interactive sensing system could accurately control the jumping and ducking motions of the character according to the corresponding sensor commands with minimal delay and error. This demonstration highlights the potential of 3D printed piezoelectric sensors as an intuitive and wearable interface for next‐generation human‐machine interaction.

These 3D printed PVDF‐TrFE sensors can function as passive tactile sensors, offering enhanced user agility compared to bulky commercial mechanical wearable sensors. Their flexibility and lightweight nature make them compelling candidates for robotic skin applications, enabling seamless integration into wearable systems and soft robotics. As a proof‐of‐concept demonstration, a nitrile glove embedded with five 3D printed sensors was used to detect external touches, with five off‐the‐shelf LED chips serving as indicators to light up when a corresponding sensor was pressed (Figure 4E; Movie S2). This simple yet effective demonstration highlights the feasibility of using 3D printed piezoelectric sensors for real‐time tactile sensing in next‐generation electronic skin.

### 2.5 Flexible Hybrid Electronics

**FIGURE 5:**
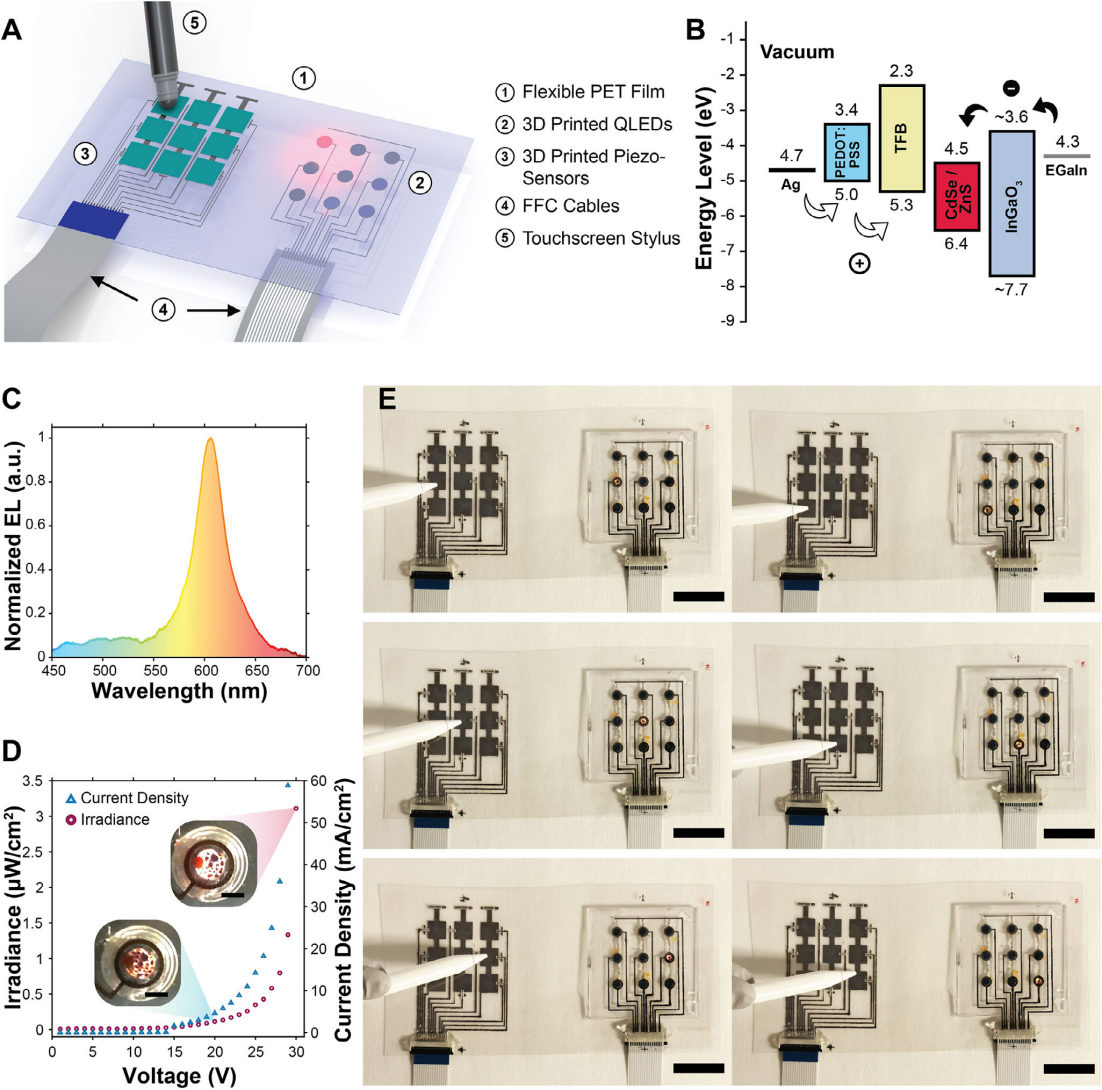
3D printed multifunctional hybrid electronics. (A) Illustration of a 3D printed multifunctional hybrid device integrating piezoelectric sensor and QLED arrays on a flexible PET substrate (piezo‐sensors indicate piezoelectric sensors). (B) Energy band diagram of the QLED showing charge carrier transport and recombination under an external voltage. Energy levels of indium–gallium oxide were estimated. (C) Normalized electroluminescence (EL) spectrum of the 3D printed QLED, with an emission peak at 606 nm and a full width half maximum (FWHM) of 34 nm (a.u. indicates arbitrary unit). (D) Current density and irradiance of the 3D printed QLED as a function of supply voltage. Irradiance was measured at a target wavelength of 600 nm. The insets show photographs of the QLED under 20 V (left) and 30 V (right) input voltages. Images were captured with a 1‐second shutter time (scale bars = 2 mm). (E) Photographs of the 3D printed multifunctional hybrid device demonstrating the activation of individual QLEDs in response to the corresponding piezoelectric sensors being pressed with a touchscreen stylus (scale bars = 20 mm).

Finally, to demonstrate 3D printing as a versatile fabrication tool, we developed a fully 3D printed, multifunctional hybrid device that integrated piezoelectric sensors and QLEDs onto a single flexible PET film (Figure 5A). As a proof‐of‐concept demonstration, we showcased real‐time control of a 3 × 3 QLED array using individual signals from a corresponding 3 × 3 PVDF‐TrFE sensor array. Movies S3 and S4, as well as Figure S15 demonstrate the DIW fabrication process of this specifically designed multifunctional hybrid device. To achieve real‐time control, the PVDF‐TrFE sensor array of the hybrid device was connected to an external microcontroller unit (MCU), which controlled the switching operation to each QLED unit through a 15‐pin flat flexible cable (FFC). A second FFC provided power to the QLED array from an external power source. See Figure S16 for a detailed circuit diagram. Notably, the QLEDs fabricated in this study were based on previous work from our group [10], with minor modifications to the layer structure to achieve improved energy band alignment (Figure 5B). The resulting devices exhibited ultra‐narrow emission spectra (Figure 5C), a typical LED *I*–*V* response, and current‐dependent electroluminescence (Figure 5D; see Figure S17 for data in negative voltages). The electroluminescent QLED array serves as an ideal test case to highlight the capabilities of the DIW‐based 3D printing technique, as its fabrication involves handling multiple functional materials and requires high precision controllability to avoid defects in the active layers. This demonstration underscores the versatility and adaptability of 3D printing, showcasing its ability to precisely handle diverse materials while maintaining tight process control. This renders it a compelling alternative for multifunctional printed electronics, avoiding the multiple cycles of material deposition, photolithography, and etching typically required by conventional methods while achieving a similar level of material integration.

Figure 5E shows a successful demonstration of the hybrid electronic system, in which QLEDs light up in response to the activation of the corresponding 3D printed piezoelectric sensors. Moreover, Movie S5 demonstrates that all nine QLEDs can be individually activated in real‐time by pressing their corresponding sensors with a touchscreen stylus pen. It should once again be noted that the QLEDs in this demonstration were powered by an external power supply, rather than by energy harvested from the 3D printed PVDF‐TrFE sensors, because the instantaneous current output (in the nanoampere range) is not yet sufficient to drive QLED operation, which typically requires currents in the microampere range. As such, the role of the piezoelectric sensors was to detect tactile stimulation and signal the MCU, which in turn controlled the ON/OFF switching between the power supply and the QLED array. Future work could explore the feasibility of a fully self‐powered system by incorporating a 3D printed power‐storage capacitor unit, potentially enabling piezoelectric‐powered QLEDs for more autonomous and integrated electronic platforms.

## 3 Discussion

In summary, we have successfully demonstrated a 3D printing workflow for the fabrication of high performance piezoelectric polymeric transducers using PVDF‐TrFE functional inks. Through systematic experimentation, we optimized both the printing parameters and electrical poling conditions to maximize the piezoelectric responses of the 3D printed devices. A key finding of this work is the strong correlation between the intrinsic coercive field of the material and the required electrical poling field needed to effectively induce piezoelectricity. Although this relationship has long been recognized in the study of inorganic piezoelectric materials [60], it has often been overlooked in more recent 3D printing work using organic piezoelectric materials. By applying a high electric field of 150 MV·m^−1^, which is more than twice the measured coercive field of PVDF‐TrFE (∼58 MV·m^−1^), we achieved substantial enhancement in piezoelectric performance with poling durations as short as 5 s. This finding is consistent with the results of Ducrot et al., who found the poling field to be effective beyond 50 MV·m^−1^ and saturated at around 100 MV·m^−1^ [20]. Furthermore, as summarized in Table 2, our optimized electrical poling treatment resulted in performance comparable to prior studies, many of which required poling durations lasting several minutes or even hours. From a commercial manufacturing perspective, a 5‐second poling duration offers significant advantages in enabling high‐throughput processing. In addition, the proposed 3D printing approach offers clear benefits over conventional fabrication techniques in both research and industrial settings. For instance, it greatly reduces material waste compared to spin‐coating, which typically results in over 95% material loss [61]. It also offers greater ease of manufacturing compared to electrospinning, which demands more complex hardware and additional equipment for electrode fabrication.

We also estimated the piezoelectric coefficient, *d_31_*, of the PVDF‐TrFE devices based on the data measured from actuation and sensing modes. We observed strong agreement between the piezoelectric coefficients acquired through characterization results from these two different methods, indicating the robustness and credibility of our experimental approaches. Utilizing the data from the DMA measurement, we were able to extract the energy harvesting capability of a 3D printed standalone PVDF‐TrFE transducer. To demonstrate the potential application of the 3D printed piezoelectric sensors, we presented three proof‐of‐concept examples targeting next‐generation wearable tactile sensing systems and flexible hybrid electronics. In the first application, a disposable rubber glove embedded with 3D printed piezoelectric sensors was used as an interactive gaming interface for a 2D jumping game, achieving high‐accuracy human‐machine interactions. In the second application, a similar type of glove embedded with five 3D printed tactile sensors was used to detect external touch stimuli, showcasing the potential for scalable, flexible, and responsive electronic skin. Lastly, we developed a fully 3D printed multifunctional hybrid device integrating piezoelectric sensors and QLEDs, which highlights the versatility of DIW‐based 3D printing as a powerful technique for multi‐material integration and to manufacture cutting‐edge flexible smart devices.

Our optimized 3D printing and poling workflow can be readily adapted to other PVDF‐based polymers, unlocking broad application potential for multifunctional electronic systems. The inherent piezoelectric sensing and actuation capabilities render these materials well‐suited for haptic VR transducers. For example, wearable VR gloves fabricated with these transducers could offer greater agility than existing bulky mechanical systems while enabling precise finger‐tracking for applications such as remote surgery. Moreover, scaling the number of sensor nodes can facilitate next‐generation robotic skins with high spatial sensitivity, enabling humanoid or industrial robots to perceive touch stimuli and safely interact with humans. Beyond pressure sensing and actuation, PVDF‐based polymers exhibit multifunctionality that extends to temperature sensing, memory storage, and even computing components [62]. Ferroelectric non‐volatile memory (FeRAM) and ferroelectric transistors (FeFETs) based on PVDF present promising prospects for flexible memory and processing units, respectively. Ultimately, this streamlined fabrication and optimization of PVDF‑based transducers could leverage the inherent multifunctionality of these advanced polymers to enable integrated electronic systems with minimal processing steps.

## 4 Conclusions

High performance organic piezoelectric transducers were fabricated using DIW‐based 3D printing of PVDF‐TrFE combined with optimized electrical poling. The piezoelectric functional layer was directly printed and electrically treated on flexible substrates, eliminating the need for transfer steps. This streamlined workflow enabled conformally attached transducers that exhibited excellent piezoelectric response, validated through both direct and inverse measurements. These 3D printed flexible transducers were integrated into wearable sensing systems, including a VR glove for human‐machine interaction and an electronic skin for real‐time tactile feedback. Furthermore, the co‐fabrication of PVDF‐TrFE sensors with QLED arrays on a single substrate demonstrated the potential of this approach for multifunctional device integration.

Future research will focus on the following aspects to both further improve these devices and utilize the developed workflow for new device types. (1) Increasing sensor node density and spatial resolution for more powerful and dynamic wearable human‐machine interfaces and electronic skins; (2) enhancing energy harvesting performance by leveraging both triboelectric and piezoelectric effects via fillers or surface modifications; (3) developing poling‐assisted DIW printing to eliminate post‐printing poling while ensuring reproducibility through comprehensive characterization and systematic optimization; (4) tuning mechanical properties by incorporating softer polymers like polydimethylsiloxane (PDMS) for improved tissue compatibility; and (5) expanding the workflow to integrate a broader range of 3D printed functional devices, and potentially incorporating bioprinted components to develop intelligent electronic‐biological interfaces. We envision that our DIW‐based 3D printing workflow for integrated electronics serves as a critical foundation for next‐generation flexible multifunctional electronics, including fully foldable smartphones, gaming consoles, E‐textiles, and beyond, where touchscreens, displays, sensors, memory, battery, and processing units are all additively printed into a single flexible platform.

## 5 Experimental Section

### 5.1 Ink Preparation

PVDF (15 wt.%) and PVDF‐TrFE (70/30 molar ratio, 15 wt.%) solutions were prepared by mixing PVDF powder (M_w_ ∼ 534000; Sigma–Aldrich) and PVDF‐TrFE powder (PolyK Technologies LLC, State College, PA, USA), respectively, in a dual‐solvent system composed of N,N‐dimethylformamide and acetone in a 4:6 volume ratio. The solutions were stirred at 700 rpm on a heated plate maintained at 80°C for 1 h to facilitate initial dispersion. Afterward, the heated plate was turned off and allowed to cool to room temperature, while stirring continued at 700 rpm for an additional 24 h.

In a nitrogen‐filled glove box, poly(9,9‐dioctylfluorene‐alt‐N‐(4‐sec‐butylphenyl)‐diphenylamine) (TFB) powder (Sigma–Aldrich, Milwaukee, WI, USA) was mixed in chlorobenzene and stirred at 700 rpm for at least 24 h to prepare a TFB chlorobenzene solution (1.5 wt.%). For device fabrication, the solution was subsequently diluted to 1/10th of its initial concentration. All other inks, including CdSe/ZnS core‐shell quantum dot (QD), were used as received from the vendors without further modification.

### 5.2 Piezoelectric Devices Fabrication

Three types of substrates were used in this work: the thin stainless‐steel sheet (0.1 mm thick, McMaster‐Carr), the rubber nitrile glove (PURPLE NITRILE* Exam Gloves, O&M Halyard Inc., Alpharetta, GA, USA), and the PET film (ZHluja 1 mil, Geheng Yongjia Technology, Shenzhen, China). Prior to the fabrication procedures, all types of substrates were cleaned with isopropanol, rinsed with deionized water, and dried using compressed air. Stainless‐steel sheets could simultaneously serve as the substrates and bottom electrodes, while nitrile rubber substrates were coated with a thin layer (100 nm) of gold using a sputterer (AJA International Inc., Hingham, MA, USA) to serve as bottom electrodes. For PET films, AgNPs (Silverjet DGP‐40LT‐15C, ANP Co Ltd, Elizabethtown, KY, USA) were printed to serve as bottom electrodes.

Active piezoelectric inks, including PVDF and PVDF‐TrFE, were degassed by spinning at 2200 rpm for 5 min in a mixer (ARE‐310, Thinky USA, Laguna Hills, CA, USA) to remove air bubbles before printing onto the bottom electrodes. The printed active inks were then dried at 80°C for 10 min. Subsequently, top electrodes were fabricated by printing AgNPs onto the active layers for nitrile rubber and PET film substrates, while Ag pastes (Silver Paste DGP80, ANP Co Ltd, Elizabethtown, KY, USA) were used for samples with stainless‐steel substrates. Fast‐curing silver epoxy (8331D, MG Chemicals, Ontario, Canada) was then applied to establish electrical connections between the electrodes and an external data acquisition unit (NI myDAQ, National Instruments, Austin, TX, USA) or a microcontroller (Arduino Micro, Arduino LLC, Boston, MA, USA).

Additionally, a standalone 3D printed PVDF‐TrFE thin film was specifically designed for dynamic mechanical analysis (DMA). Two layers of PVDF‐TrFE functional ink were deposited onto a stainless‐steel substrate coated with a thin layer of silicone oil and dried at 80°C for 10 min. The thin film was manually removed from the substrate and cleaned with ethanol. After cleaning, gold electrodes (100 nm thick) were sputtered onto both surfaces using the AJA sputterer, forming an active region of 40 mm × 10 mm × 21 µm. The standalone sample was subsequently poled at + 150 MV·m^−1^ for 5 s. Silver epoxy was applied as a conductive adhesive to attach copper enameled wires to gold electrodes, which facilitated electrical connections to the NI myDAQ.

### 5.3 QLED Fabrication

PET films were cleaned using the same procedure described previously. AgNPs were printed on the substrate to serve as the anode. PEDOT:PSS (0.8 wt.%, Ossila, Sheffield, UK), TFB (0.15 wt.%), and QD toluene (10 mg mL^−1^, target emission at 600 nm, from NNCrystal US Corporation, Fayetteville, AR, USA) solutions were filtered through a 0.47 µm filter and sequentially deposited onto the substrate in the stated order. Room temperature vulcanizing silicone (Loctite SI 595 CL, Henkel Corp., Stamford, CT, USA) was printed around the active layers to provide structural stability to the top electrode. Following this step, eutectic gallium‐indium (EGaIn; Sigma–Aldrich, St. Louis, MO, USA) was deposited as the cathode, and EGaIn reconfiguration was carried out [11]. Silver epoxy was applied to connect the cathode to an external power supply. Finally, the device was encapsulated with PDMS (Sylgard 184, Dow, Midland, MI, USA) to enhance its durability and environmental stability.

Both the piezoelectric devices and QLEDs were fabricated using a custom‐built 3D printing system comprising a 3D gantry system (ANT 130, Aerotech Inc., Pittsburgh, PA, USA) and a pneumatic pressure regulator (Ultimus V, Nordson EFD, Westlake, OH, USA). Detailed printing and curing parameters for the materials mentioned above are summarized in Table S1.

### 5.4 FT‐IR Measurement

FT‐IR scans of the 3D printed PVDF and PVDF‐TrFE films (10 mm × 10 mm, without electrodes) were conducted using Nicolet iS50 Spectrometer (Thermo Fisher Scientific, Waltham, MA, USA), which was equipped with a built‐in diamond attenuated total reflection (ATR) unit and a DLaTGS detector.

### 5.5 Thickness and Width Measurements

For each of the different printing pressures (25 kPa, 35 kPa, 45 kPa, and 55 kPa) and printing speeds (100, 200, 300, 400, and 500 mm min^−1^), four lines of PVDF‐TrFE functional ink (15 wt.% concentration) were 3D printed on cleaned glass substrates with 25‐gauge stainless‐steel nozzles (Nordson EFD, Westlake, OH, USA) and dried at 80°C for 10 min. The 2D profiles of the printed lines under each printing parameter were measured (Figure S2) using a surface profilometer (P‐10, KLA‐Tencor, Milpitas, CA, USA).

### 5.6 P‐E Measurement and Poling

The electrodes of the 3D printed piezoelectric samples were connected to a Sawyer‐Tower circuit, as illustrated in Figure S3. To obtain *P‐E* curves, bipolar sinusoidal voltages equivalent to ±350 MV·m^−1^ at 100 Hz were applied for 0.1 s. For poling purposes, unipolar sinusoidal voltages (at desired electric fields and durations) were applied at 100 Hz. In this work, we defined 100 cycles as equivalent to 1 s of poling.

### 5.7 3D Printed Bender Actuation Measurement

The 3D printed piezoelectric benders were installed in a cantilever configuration, with electrodes connected to the positive and negative terminals of the high‐voltage supply, consisting of a function generator and a high‐voltage amplifier. A laser‐based proximity sensor (LK‐H022, Keyence Corporation of America, Itasca, IL, USA) with a measurement resolution of 0.01 µm and a sampling frequency of 1 kHz was used to record the displacement of the 3D printed piezoelectric bender at a designated target point (28 mm from the root of the cantilever, center point of width).

### 5.8 Storage Conditions

The 3D printed PVDF‐TrFE benders used in the 60‐day aging test were stored under ambient laboratory conditions at room temperature (ca. 27°C), without control over humidity or temperature. The samples were not subjected to continuous mechanical loading or additional electrical poling between measurements.

### 5.9 3D Printed Bender Sensor Measurement

A high‐precision *z*‐axis linear stage (ANT130L‐ZS, Aerotech Inc., Pittsburgh, PA, USA) equipped with an empty syringe and nozzle was programmed to apply controlled deformation to a cantilever‐like PVDF‐TrFE sensor, which was fabricated, poled, and installed similarly to its bender counterparts. To eliminate triboelectric signal interference, the syringe nozzle was wrapped in an insulating layer (Parafilm M, Bemis Mfg, Neenah, WI, USA). The sensor electrodes were connected in parallel with a 10 MΩ resistor, which converted the piezoelectric‐induced currents into voltage signals, subsequently processed by the DAQ. The controlled deformation parameters were displacement: ±1 mm, speed: 500 mm min^−1^, and number of cycles: 10.

### 5.10 DMA Measurement

The RSA‐G2 DMA system (TA Instruments, New Castle, DE, USA) was used to apply user‐defined cyclic strains (±0.1%, ±0.3%, ±0.5% at 1 Hz) to the PVDF‐TrFE standalone thin film for a duration of 100 s (100 actuation cycles). The sample was secured in two clamps attached to the DMA and pre‐strained by 1 mm prior to testing (Figure S7). Additionally, the analog output port of the RSA‐G2 provided real‐time strain data as voltage signals. These strain signals were fed into the NI myDAQ alongside the real‐time electrical signals generated by the piezoelectric sample.

### 5.11 QLED Characterization

The current‐voltage (*I*–*V*) characteristics of the QLEDs were measured using a precision measurement power supply (Keithley 2280S‐32‐6, Tektronix, Beaverton, OR, USA) operated in controlled voltage mode. DC voltages were supplied in incremental steps of 1 V, starting from 1 to 30 V, and the corresponding current response was recorded by the instrument. Simultaneously, irradiance values at different voltages were collected using a photodiode sensor (S130V, Thorlabs Inc., Newton, NJ, USA), calibrated for a target wavelength of 600 nm. Additionally, at a driving voltage of 30 V, the emission spectrum of a QLED was captured using a spectrometer (Flame, Ocean Insight Inc., Orlando, FL, USA).

### 5.12 Python Code

A modified version of an open‐source (MIT licensed) Python‐based 2D jumping game was used to demonstrate the sensor interface (see Movie S1). Custom game assets (e.g., player character and background images) were redesigned to better reflect the context of our application. The original game code was developed by Jiahong‐Guan, sourced from GitHub [https://github.com/Jiahong‐Guan/chrome‐dino‐python.git].

### 5.13 Wearable Piezoelectric Sensors

Daniel Wai Hou Ng consented to wear the device during the experiments. Approval from an ethics committee is not required.

### 5.14 Statistical Analysis

All experimental data including error bars are represented as the mean ± standard deviation. The sample size (*n*) for experimental data was included in the figure captions and main text.

## Conflicts of Interest

M.C.M. serves on the Scientific Advisory Board and holds equity in GRIP Molecular Technologies. M.C.M. is cofounder and CSO of Flui3D Inc. These interests have been reviewed and managed by the University of Minnesota in accordance with its Conflict of Interest policies. The authors declare that they have no other competing interests.

## Supporting information




**Supporting file 1**: smll72210‐sup‐0001‐SuppMat.docx.

**Supporting file 2**: smll72210‐sup‐0002‐MovieS1.mp4.

**Supporting file 3**: smll72210‐sup‐0003‐MovieS2.mp4.

**Supporting file 4**: smll72210‐sup‐0004‐MovieS3.mp4.

**Supporting file 5**: smll72210‐sup‐0005‐MovieS4.mp4.

**Supporting file 6**: smll72210‐sup‐0006‐MovieS5.mp4.

## Data Availability

The data that support the findings of this study are openly available in Data Repository for U of M (DRUM) at https://doi.org/10.13020/67k0‐bg27.
