# Supplementary material for: 3D Printed Flexible Piezoelectric Sensors for Integrated Hybrid Electronics
Source: Small. 2025 Dec 31;22(11):e11146. doi: 10.1002/smll.202511146 (PMC12921551; doi:10.1002/smll.202511146)
Supplement: Supplementary file 1 — Supporting file 1: smll72210‐sup‐0001‐SuppMat.docx. [file SMLL-22-e11146-s004.docx]

Supporting Information

**3D Printed Flexible Piezoelectric Sensors for Integrated Hybrid Electronics**

*Daniel Wai Hou Ng, Hassene Ben Atitallah, Ghazaleh Haghiashtiani, Jinsheng Fan, Hyunjun Kim, Guebum Han, Riyan Mendonsa, Razman Zambri, Michael C. McAlpine**

Daniel Wai Hou Ng, Jinsheng Fan, Hyunjun Kim, Guebum Han, and Michael C. McAlpine Michael C. McAlpine

Department of Mechanical Engineering, University of Minnesota, Minneapolis, Minnesota 55455, United States

E-mail: mcalpine@umn.edu

Hassene Ben Atitallah, Ghazaleh Haghiashtiani, Riyan Mendonsa, and Razman Zambri

Seagate Technology LLC, Bloomington, Minnesota 55435, United States.

**Experimental Details**

*1. Rheology measurement*

PVDF (15 wt%) and PVDF-TrFE (70/30 molar ratio, 15 wt%) solutions were prepared by mixing PVDF powder (Mw ~ 534000; Sigma-Aldrich) and PVDF-TrFE powder (PolyK Technologies LLC, State College, PA, USA), respectively, in a dual-solvent system composed of N,N-dimethylformamide (DMF) and acetone in a 4:6 volume ratio. The Discovery HR-20 rheometer (TA Instruments, New Castle, DE, USA) was used to measure the viscosity of each functional inks.

*2. Thickness & width measurements*

For each of the different printing pressures (25 kPa, 35 kPa, 45 kPa, and 55 kPa) and printing speeds (100 mm min^-1^, 200 mm min^-1^, 300 mm min^-1^, 400 mm min^-1^, and 500 mm min^-1^), four lines of PVDF-TrFE functional ink (15 wt% concentration) were 3D printed on cleaned glass substrates with 25-gauge stainless-steel nozzles (Nordson EFD, Westlake, OH, USA) and dried at 80 °C for 10 minutes. The 2D profiles of the printed lines under each of the printing parameters were measured (Figure S2) using a surface profilometer (P-10, KLA-Tencor, Milpitas, CA, USA). A custom MATLAB script was developed to compute the average thickness and width of the printed lines.

*3. Materials characterization*

*3.1 Fourier-transform infrared spectroscopy (FT-IR)*

FT-IR scans of the 3D printed PVDF and PVDF-TrFE films (10 mm × 10 mm dimensions, without electrodes) were carried out using the Nicolet™ iS50 Spectrometer (Thermo Fisher Scientific, Waltham, MA, USA), equipped with a built-in diamond attenuated total reflection (ATR) unit and a DLaTGS detector.

*3.2 Ferroelectric characterization*

A Sawyer-Tower circuit^[1]^ (Figure S3) was constructed to obtain the polarization-versus-electric field (*P-E*) curve of the 3D printed PVDF and PVDF-TrFE films.

A high-voltage amplifier (TREK 609C, Advanced Energy Industries Inc., Denver, CO, USA) was connected to a signal generator (DG1022, RIGOL Technologies, Suzhou, China) capable of producing programmable voltage waveforms. This combined setup served as a high-voltage supply. The positive and negative terminals of the high-voltage amplifier were connected in series with the ferroelectric sample and a high-voltage-rated capacitor (R76UN23304040J, KEMET Corporation, Fort Lauderdale, FL, USA). A multi-channel oscilloscope (MSO5104, RIGOL Technologies, Suzhou, China) recorded voltage responses at two key points in the circuit. Additionally, custom MATLAB scripts were developed to enable remote control of the signal generator and oscilloscope to ensure safe operation.

To obtain the P-E plot of the ferroelectric sample, a bipolar sinusoidal voltage at 100 Hz was applied. The electric field (*E*, *x*-axis) and polarization (*P*, *y*-axis) values for the plot were computed using **Equation S1** and **S2**:

$$\begin{aligned} x=E=\frac{V_{1}}{t_{film}} \#\left( S1 \right) \end{aligned}$$

$$\begin{aligned} y=P=\frac{C_{ref}}{A_{film}}\cdot V_{2} \#\left( S2 \right) \end{aligned}$$

Here, *t_film_* is the thickness of the ferroelectric film (m), *C_ref_* is the capacitance of the reference capacitor (F), *A_film_* is the active area of the ferroelectric film where the electric field is in effect (m^2^), and *V_1_* and *V_2_* are the measured voltage signals (V).

*4. Electrical poling*

Electrical poling was performed using the Sawyer-Tower circuit described earlier. After the direct-ink-writing (DIW) fabrication of a piezoelectric sample, its capacitance at 1 kHz was measured using an LCR meter (ZM2372, NF Corporation, Yokohama, Japan). The thickness of the piezoelectric sample was then estimated using the following **Equation S3**:

$$\begin{aligned} t_{piezo}=\frac{\epsilon_{r}\cdot\epsilon_{0}\cdot A_{piezo}}{C_{piezo}} \#\left( S3 \right) \end{aligned}$$

Here, *t_piezo_* is the thickness of the piezoelectric film (m), *ε_r_* is the relative permittivity of the piezoelectric material, *ε_0_* is the vacuum permittivity (8.854 × 10^-12^ F m^-1^), *A_piezo_* is the active area of the piezoelectric film where electric field is in effect (m^2^), and *C_piezo_* is the measured capacitance of the piezoelectric sample at 1 kHz (F).

After determining the thickness of the piezoelectric sample, the required voltage to generate a specific electric field was calculated by multiplying the thickness by the target electric field strength. To mitigate the risk of dielectric breakdown, unipolar sinusoidal AC voltages (peak values equal to the desired electric fields) at 100 Hz were applied instead of DC voltages.

*5. Driving of 3D printed piezoelectric bender and actuation measurements*

To apply a high voltage for actuating the 3D printed PVDF-TrFE benders, the top electrode (silver paint layer) and bottom electrode (stainless-steel substrate) were connected to the positive and negative terminals of the high-voltage supply, respectively. A custom MATLAB script was developed to allow precise control over the driving voltage parameters (e.g., frequency, peak-to-peak value, duration), enabling the user to supply the required voltage on demand.

The actuation measurement setup is illustrated in Figure 2D and Figure S9. A laser-based proximity sensor (LK-H022, Keyence Corporation of America, Itasca, IL, USA) with a resolution of 0.01 µm and a sampling frequency of 1 kHz was employed to measure the displacement of the 3D printed piezoelectric bender at a designated target point. To minimize external disturbances, the setup was mounted on an optical table to isolate it from ground vibrations, and an acrylic enclosure was used to shield against airflow-induced noise.

The laser sensor was linked to a controller (LK-G5001, Keyence Corporation of America, Itasca, IL, USA), which converted sensor readings into analog signals (1 µm = 1 V). These signals were recorded using a data acquisition unit (NI myDAQ, National Instruments, Austin, TX, USA). Additionally, the TREK 609C high-voltage amplifier featured a voltage monitoring port, which outputted a step-down version of the high-voltage signal at a 1:1000 ratio. This monitoring port was connected to the same data acquisition unit, enabling simultaneous recording of driving voltage data and output actuation data. Figure S4 presents an example of a driving voltage waveform and the corresponding actuation response from the 3D printed piezoelectric bender.

*6. Measurement for 3D printed cantilever-like sensor*

A 3D gantry robot (ANT-130, Aerotech Inc., Pittsburgh, PA, USA), equipped with an empty syringe and nozzle, was programmed to apply controlled deformations to a PVDF-TrFE sensor in cantilever configuration, which was fabricated, poled, and installed similarly to its piezoelectric bender counterparts. The nozzle of the syringe was wrapped in an insulating layer (Parafilm® M, Bemis Mfg, Neenah, WI, USA) to prevent triboelectric signal interference. Moreover, to protect the active components and prevent unwanted electrical artifacts, the point of contact was chosen as the stainless-steel substrate rather than the piezoelectric layer. The two electrodes of the cantilever-like sensor were connected in parallel with a 10 MΩ resistor, which converted the piezoelectric-induced current into voltage readings that could be processed by the NI myDAQ. The controlled deformation parameters were set as follows: displacement: ± 1 mm, speed: 500 mm min^-1^, number of cycles: 10.

*7. Fabrication and DMA measurement for 3D printed standalone sensor*

To facilitate easy removal of the standalone PVDF-TrFE sample, a stainless-steel sheet coated with a thin layer of silicone oil was used as the printing substrate. Two layers of PVDF-TrFE functional ink were deposited on the substrate using previously optimized printing parameters and then dried at 80 °C for 10 minutes. After drying, a thin gold layer (100 nm) was deposited on the surface of the active layer via physical vapor deposition (PVD) sputtering (AJA International Inc., Hingham, MA, USA) to form the top electrode. The thin film, consisting of the active layer and gold top electrode, was then carefully detached from the stainless-steel substrate.

The non-electroded side of the PVDF-TrFE thin film was thoroughly cleaned with ethanol to remove any silicone oil residue. Subsequently, another 100 nm gold layer was sputtered to form the bottom electrode. The final standalone samples had dimensions of 50 mm × 10 mm × 21 µm, with an active region of 40 mm × 10 mm × 21 µm where the two gold electrodes sandwiched the piezoelectric layer. The standalone sample was subsequently poled at +150 MV m^-1^ for 5 seconds. Finally, silver epoxy (8331D, MG Chemicals, Ontario, Canada) was used as the conductive adhesive to connect the gold electrodes with copper enameled wires, which were later used to transmit electric signals to the data acquisition unit.

Note that two layers of functional ink were deposited to increase the film thickness, thereby enhancing the mechanical strength of the standalone sample and reducing the risk of plastic deformation during manual removal. Additionally, thin gold electrodes were chosen instead of 3D printed silver paint electrodes to minimize the interference of the mechanical properties of the silver paint layers with the measurement result from the dynamic mechanical analyzer (DMA). Likewise, ultra-thin enameled wires were used to transmit electric signals due to their light weight and minimal impact on the measurement result.

The RSA-G2 DMA system (TA Instruments, New Castle, DE, USA) was used to apply user-defined cyclic strains to the PVDF-TrFE standalone thin film. The sample was secured in the DMA clamps (Figure S7b) and pre-strained by 1 mm before testing. The DMA was programmed to operate at a strain rate of 1 Hz for a total duration of 100 seconds (= 100 cycles).

*8. QLED measurement*

The current-voltage (*I*–*V*) characteristics of the quantum dot light-emitting diode (QLED) were measured using a precision measurement power supply (Keithley 2280S-32-6, Tektronix, Beaverton, OR, USA) operated in controlled voltage mode. DC voltages were supplied in incremental steps, and the corresponding current response was recorded by the instrument. Simultaneously, irradiance values at different voltages were collected using a photodiode sensor (S130V, Thorlabs Inc, Newton, NJ, USA), calibrated for a target wavelength of 600 nm.

Additionally, at a DC bias of 30 V, the emission spectrum of a QLED was captured using a spectrometer (Flame, Ocean Insight Inc., Orlando, FL, USA).

*9*. *Energy band levels*

The work functions of silver nanoparticles (AgNPs) and eutectic gallium-indium (EGaIn) were adapted from the study by Kong et al.^[2]^ The energy band levels of poly(3,4-ethylenedioxythiophene):polystyrene sulfonate (PEDOT:PSS), poly(9,9-dioctylfluorene-alt-N-(4-sec-butylphenyl)-diphenylamine) (TFB), and CdSe/ZnS core-shell quantum dots (QDs) were referenced from the work of Kim et al.^[3]^

The energy band levels of gallium-indium oxide (InGaO₃) were estimated using band alignment data reported by Dive, Varley, and Banerjee, who provided valence-band offset (-2.5 eV) and conduction-band offset (+0.18 eV) values relative to cadmium telluride (CdTe).^[4]^ The energy levels of CdTe were obtained from the review by Li and Wu, where the conduction band minimum (E_CB_) and valence band maximum (E_VB_) were reported as –3.8 eV and –5.2 eV, respectively.^[5]^

Based on the reported offsets, the estimated energy levels for InGaO₃ are:

- E_CB_ = (-3.8 + 0.18) eV = -3.62 eV,
- E_VB_ = (-5.2 - 2.5) eV = -7.7 eV.


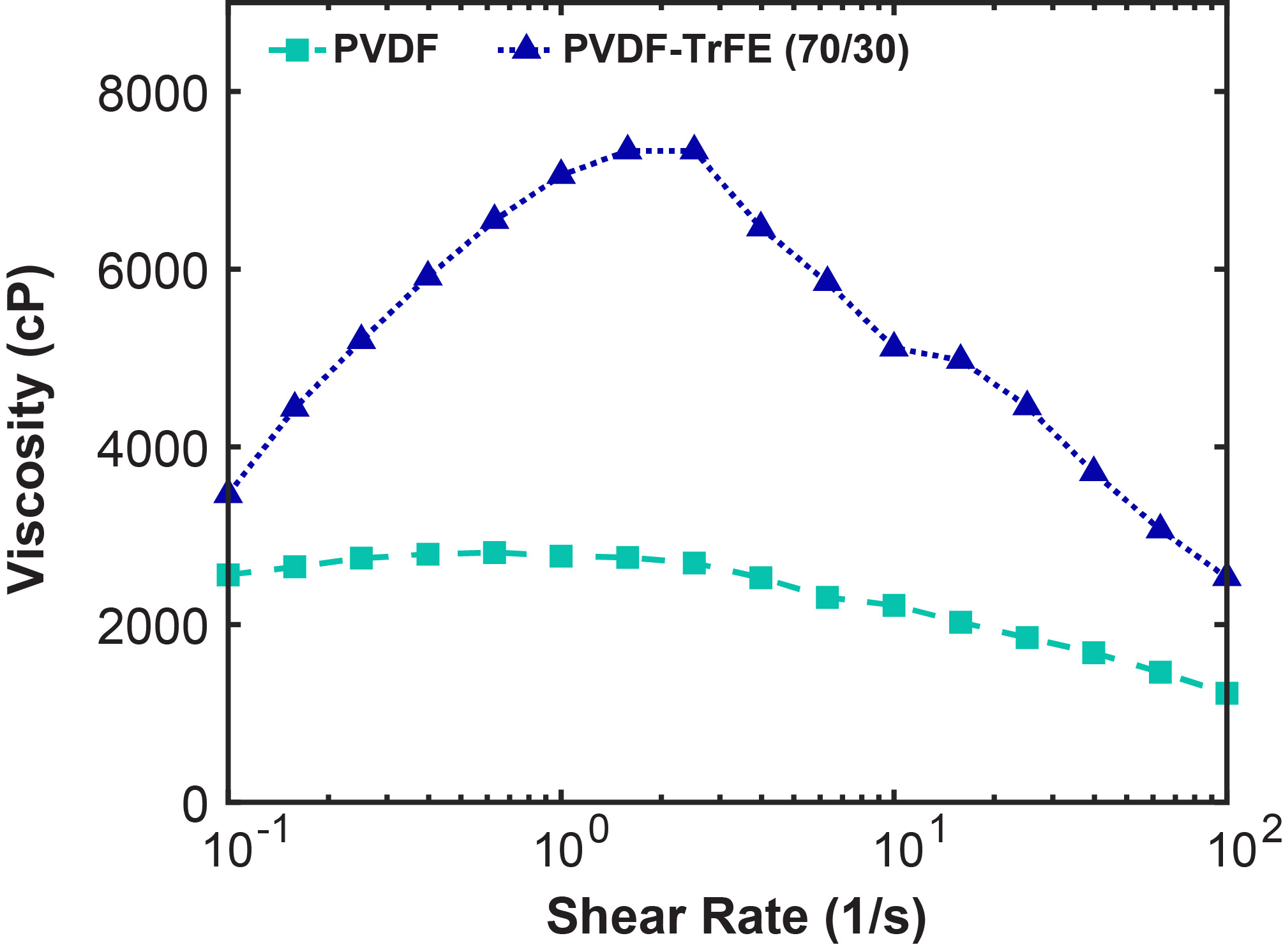


**Figure S1**.Viscosity of 15 wt% PVDF and 15 wt% PVDF-TrFE (70/30) functional inks in a mixed solvent of DMF and acetone. The measurement was carried out using the Discovery HR-20 rheometer (TA Instruments, New Castle, DE, USA).


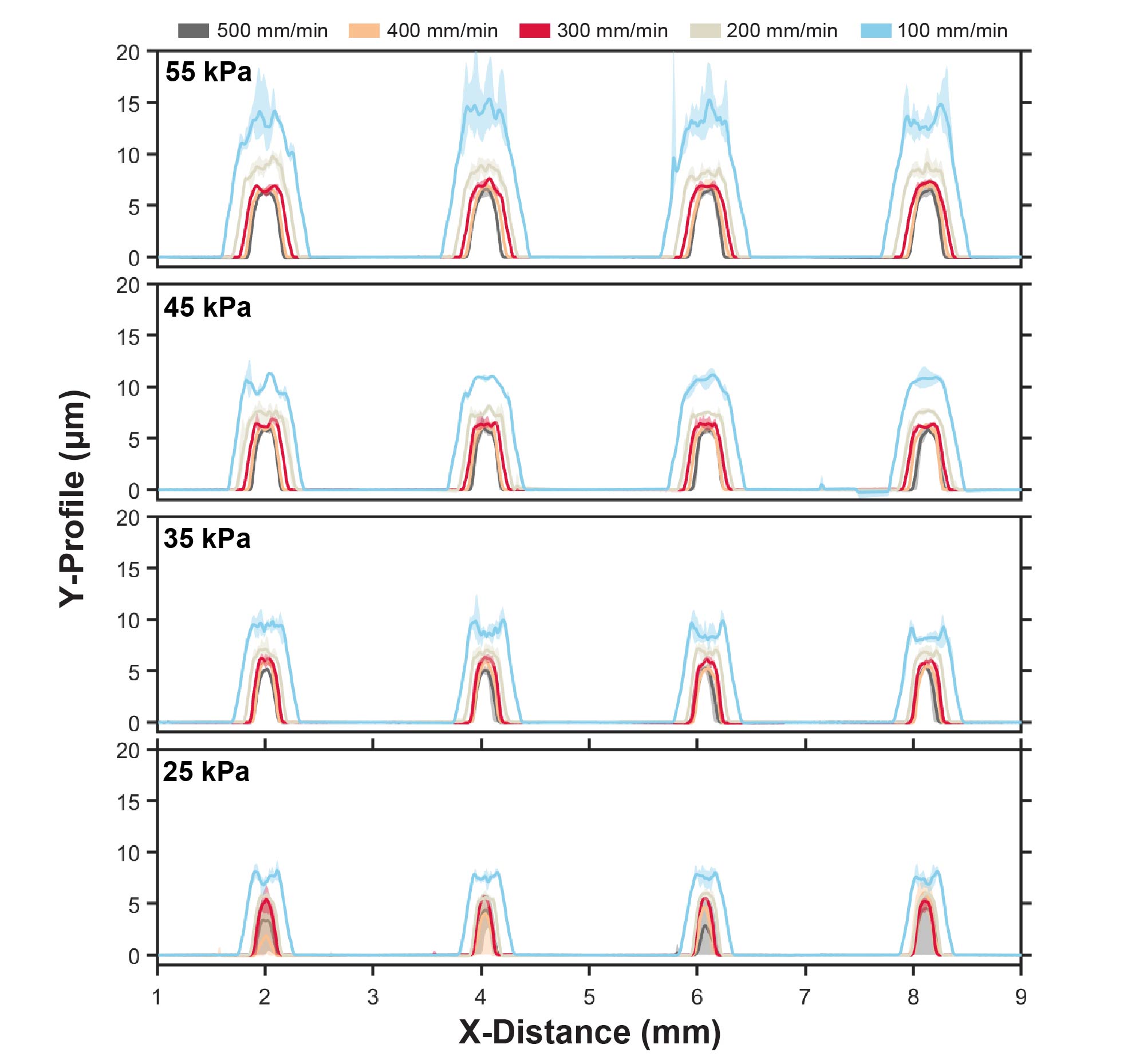


**Figure S2**. 2D profiles of 15 wt% PVDF-TrFE print lines under different printing parameters. Printing was carried out with 25-gauge stainless-steel nozzles, and all printed lines were dried at 80 °C for 10 minutes prior to measurement.


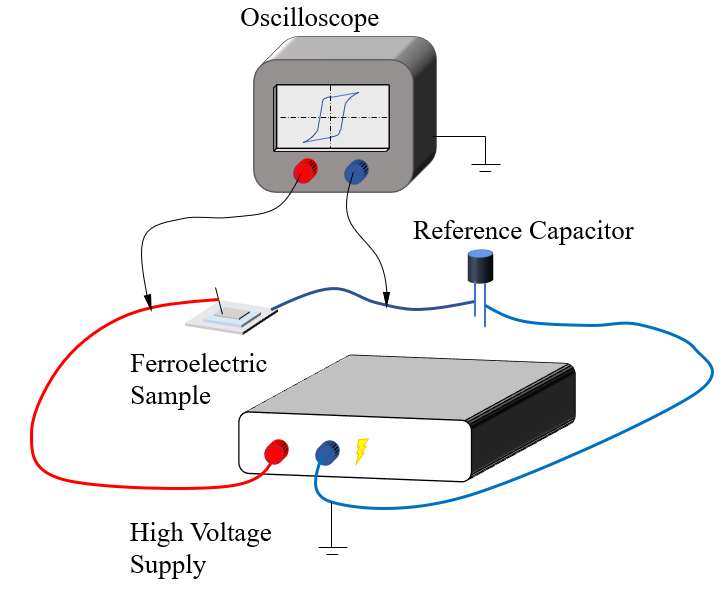


**Figure S3**. Custom-built Sawyer-Tower circuit for ferroelectric characterization and electrical poling.

Please note that all piezoelectric materials are ferroelectric; however, the converse is not true. A hysteretic *P*-*E* plot is typically indicative of ferroelectricity but not necessarily piezoelectricity. Therefore, in Figure S3, the piezoelectric sample is labeled as a ferroelectric sample for clarity.


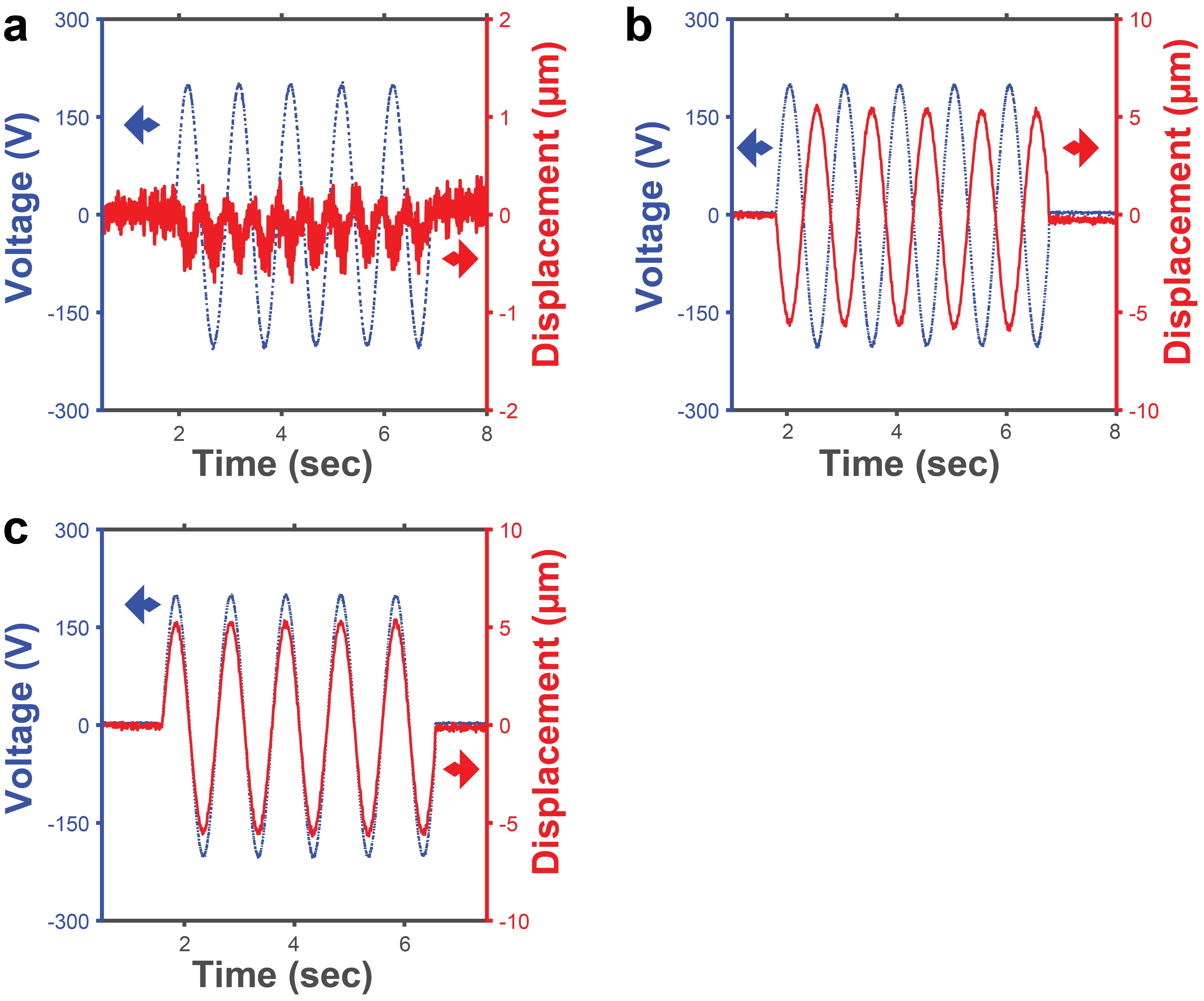


**Figure S4**. Consecutive plots demonstrating the actuation behavior of a PVDF-TrFE bender under an identical driving voltage (blue dashed lines) after the bender was subjected to a) no poling treatment, b) + 130 MV m^-1^ poling for 1 second, and c) -130 MV m^-1^ poling for 1 second.


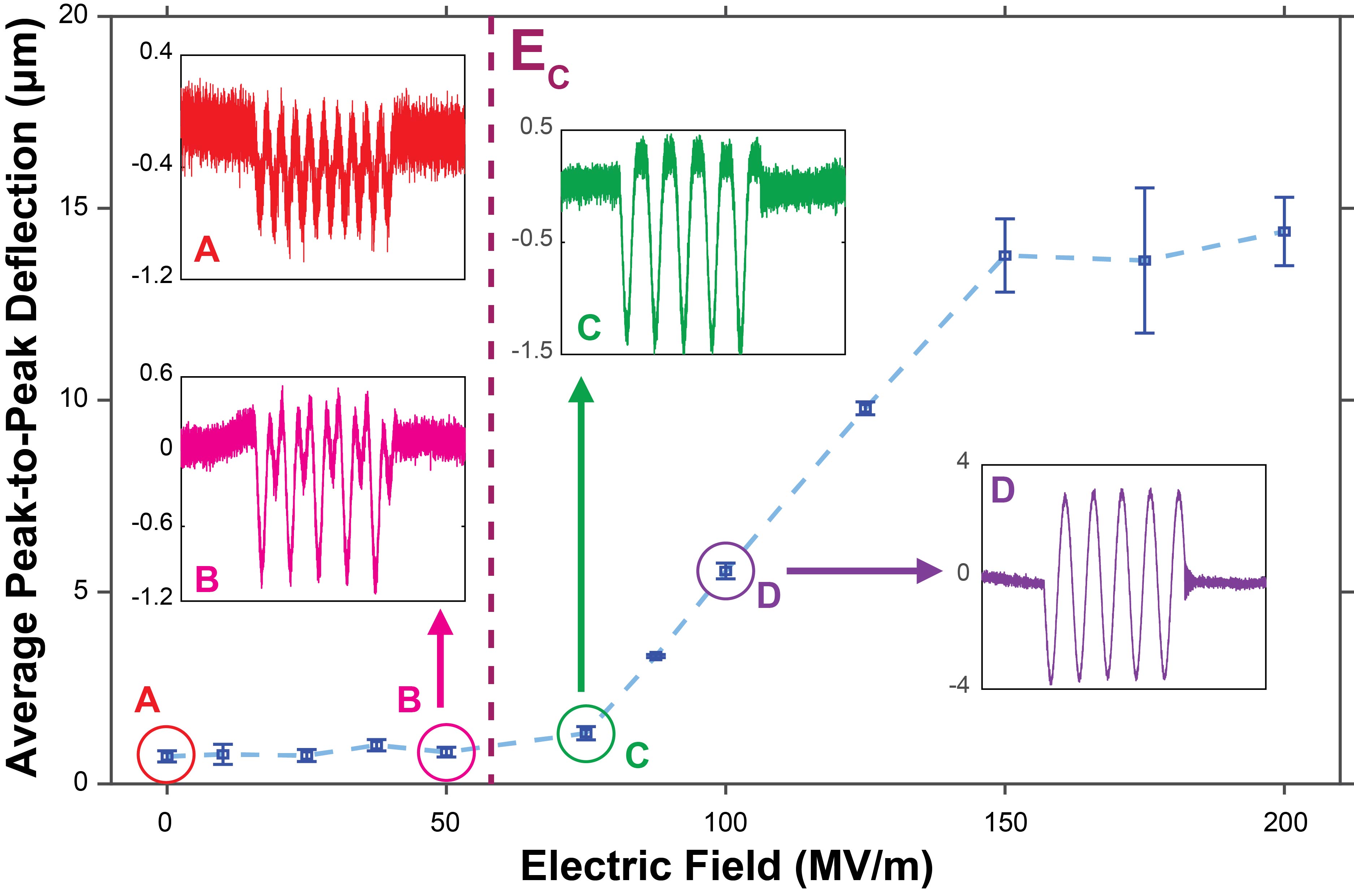


**Figure S5**. Development of piezoelectricity in the same PVDF-TrFE bender under an increasing electric field. The poling duration was fixed at 1 second. After each poling treatment, the actuation response of the bender was tested under a fixed driving voltage (sinusoidal at 1 Hz, ±250V, 5 cycles). The vertical dashed line represents the value of the coercive field for PVDF-TrFE (70/30).

The insets (A–D) display the recorded actuation behaviors at four critical poling electric fields.

- At 0 MV m^-1^ (unpoled, Inset A) – The bender exhibited unidirectional actuation regardless of the driving voltage direction, showing nearly identical magnitude for both positive and negative driving voltages.
- At 50 MV m^-1^ (Inset B) – While the actuation remained unidirectional, the bender began to exhibit polar behavior, with a greater actuation magnitude when the driving voltage was applied in a specific direction.
- At 75 MV m^-1^ (Inset C) – The polarity became more pronounced, as the bender only actuated when the driving voltage was in a specific direction, while it developed “immunity” to driving voltage in the opposite direction.
- At 100 MV m^-1^ (Inset D) – Bidirectional actuation was observed, with a strong dependence on the driving voltage direction. However, symmetry had not yet fully developed, as the actuation in the negative region remained slightly more pronounced than in the positive region. Symmetric actuation behavior was achieved at higher poling electric fields.


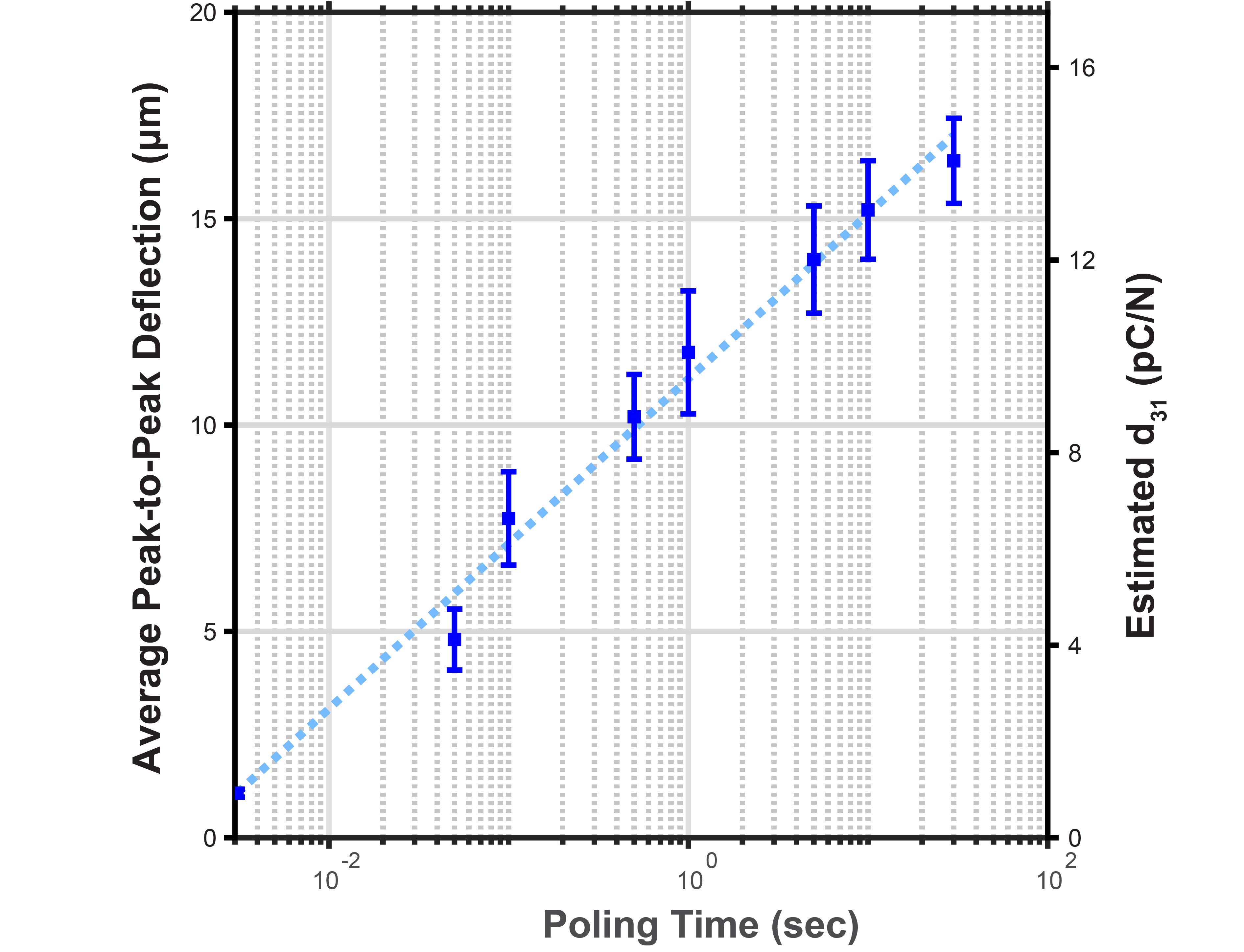


**Figure S6**. Semi-logarithmic plot for actuation of 3D printed PVDF-TrFE benders (*n* = 3) as a function of poling time (fixed poling electric field of + 150 MV m^-1^). R^2^ = 0.976.

Note that in Figure S6, the first point was not included in the computation of the linear fitting line and R^2^ value because the corresponding poling time was zero (pre-poling). When converted to a logarithmic scale, the *x*-value of the first point became -∞. Hence, a linear fitting line was first computed using the remaining data points, then a pseudo-*x*-value of the first point was extrapolated from its deflection value.

**Calculating the Piezoelectric Coefficient (*d_31_*)**

1. **Direct Piezoelectric Effect**

The piezoelectric constant *d_31_* of the PVDF-TrFE standalone sample was estimated based on data collected from a DMA using the piezoelectric constitutive **Equation S4**^[6]^ below:

$$\begin{aligned} D_{3}=d_{31}T_{1}+\epsilon^{T}E_{3} \#\left( S4 \right) \end{aligned}$$

Where:

- D_3_ is the electric charge displacement density in the thickness direction (C·m^-2^),
- d_31_ is the piezoelectric coupling coefficients in strain-charge form (C·N^-1^),
- T_1_ is the input stress along the force direction (N·m^-2^),
- ϵ^T^ is the electric permittivity of the material (F·m^-1^), and
- E_3_ is the applied electric field along the thickness direction (V·m^-2^).

Since no external electric field was applied to the PVDF-TrFE standalone sample, the second term in Equation S4 is eliminated. By definition:

*D_3_ = Q/A_piezo_* and *T_1_ = F_1_/A_cross_*

Where:

- *Q* is the electric charge generated (C),
- *A_piezo_* is the effective electrode-contact area with the piezoelectric layer (m^2^), and
- *A_cross_* is the cross-sectional area where stress is applied (m^2^).


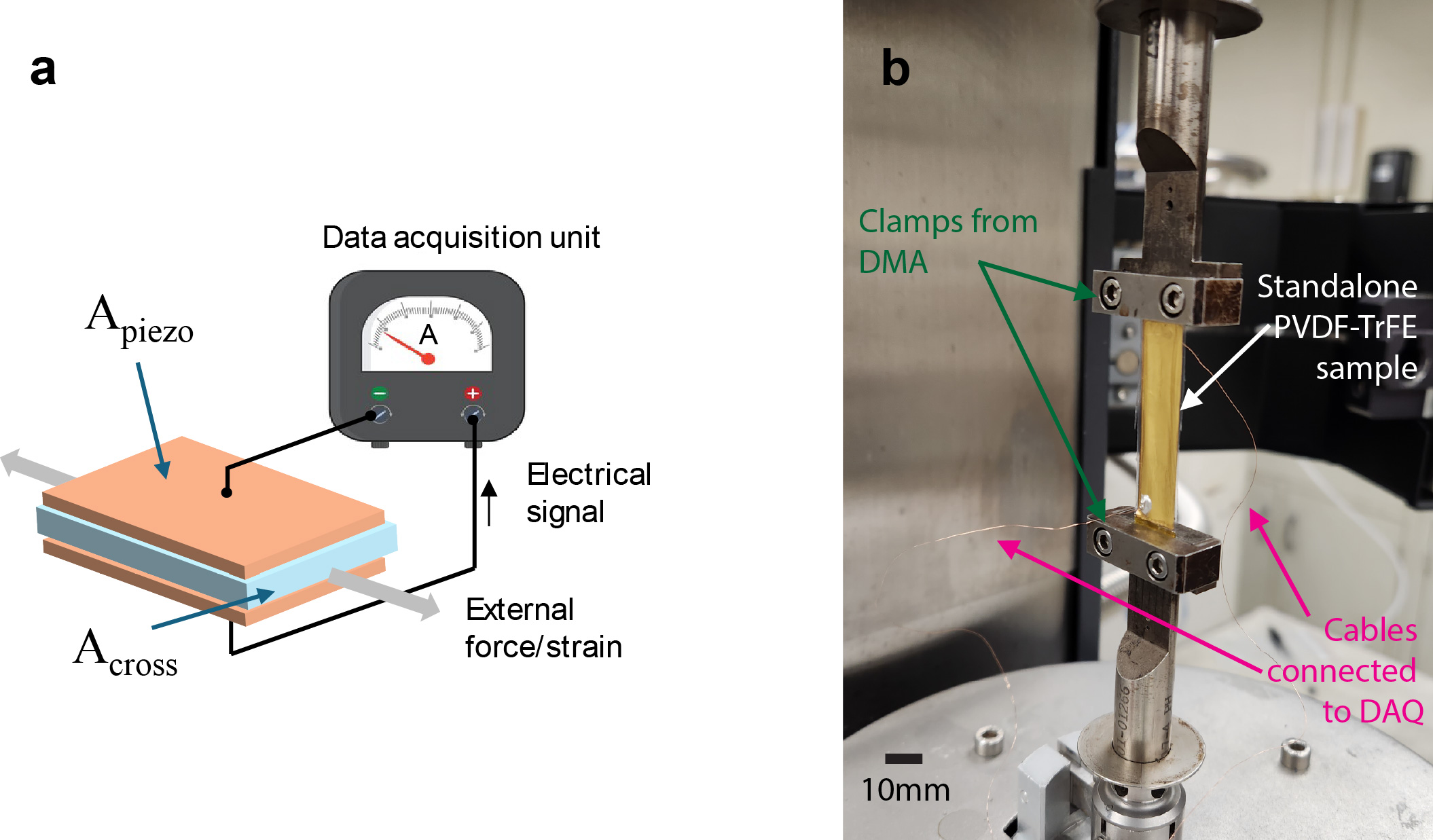


**Figure S7**. a) Schematic of direct piezoelectric effect. b) Measurement setup of the standalone PVDF-TrFE sample using RSA-G2 DMA system.

Thus, Equation S4 simplifies to:

$$d_{31}= \frac{D_{3}}{T_{1}} = \frac{Q}{l\cdot w}\times\frac{w\cdot t}{F_{1}} = \frac{t}{l}\times\frac{Q}{F_{1}}$$

Taking the time derivative yields **Equation S5**:

$$\begin{aligned} d_{31}= \frac{t}{l}\times\frac{I}{\frac{dF_{1}}{dt}} \#\left( S5 \right) \end{aligned}$$

where *I* is the measured current (A or C s^-1^). Moreover, for the standalone PVDF-TrFE sample, thickness (t) = 21 µm, width (w) = 10 mm, and length (*l*) = 41 mm.

In the experiment, the current generated from the PVDF-TrFE standalone sample and the force measured by the DMA system were recorded simultaneously using the same data acquisition unit (NI-myDAQ). Cyclic strains (0.2%, 0.6%, and 1.0% at 1 Hz) were applied for 100 seconds, and a portion of the collected data at 0.2% strain is shown in Figure S8.


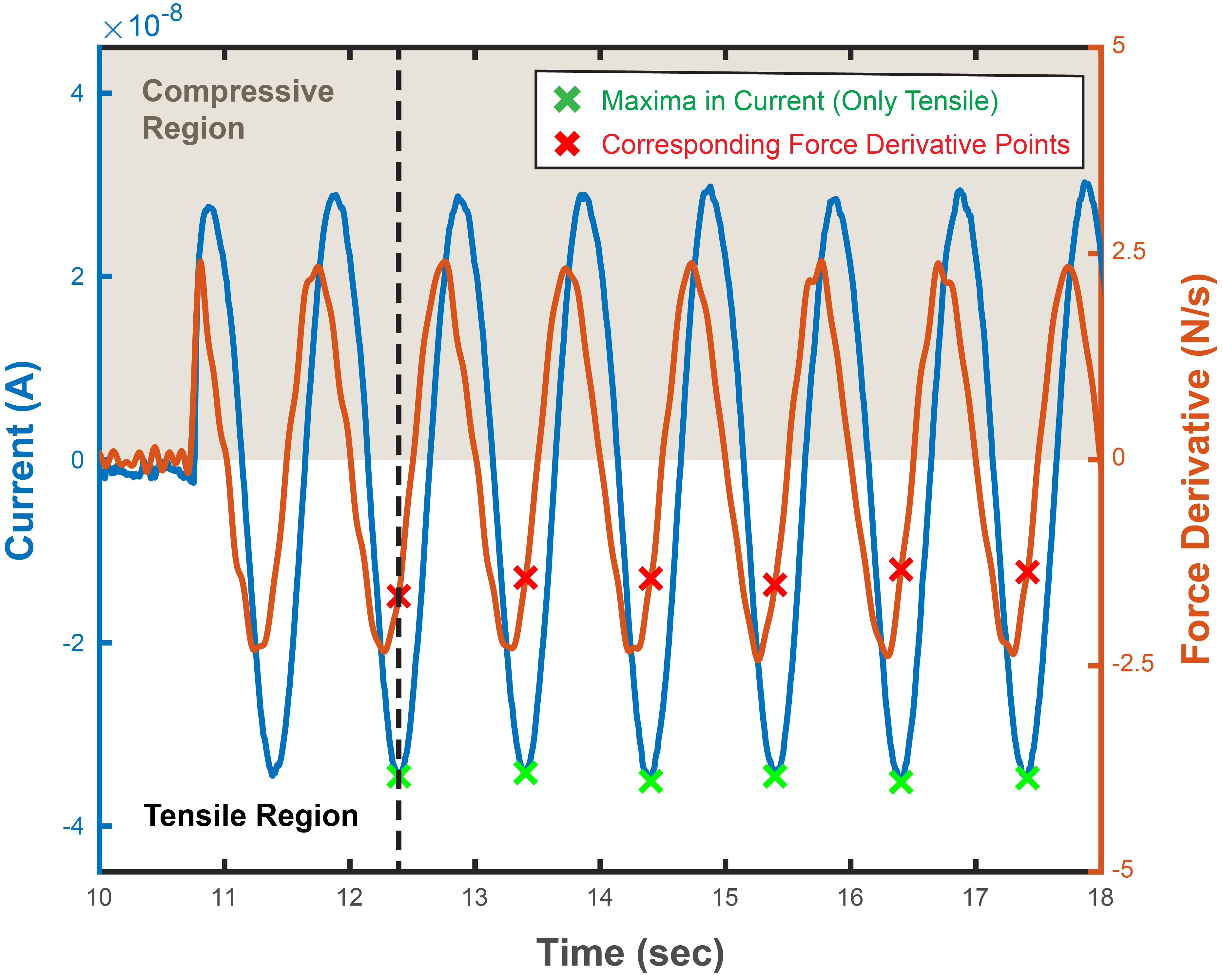


**Figure S8**. A portion of the current and force derivative signals collected from a 0.2%/sec cyclic strain experiment.

Substituting the values of the current and force derivative at the dashed line in Figure S8 into Equation S5, we present an example computation as follows:

$$d_{31}= \frac{t}{l}\times\frac{I}{\frac{dF_{1}}{dt}}$$

$$d_{31}= \frac{21\times{10}^{-6}m}{41\times{10}^{-3}m}\times\frac{-3.457\times{10}^{-8}C s^{-1}}{-1.656 N s^{-1}}$$

$$d_{31}= 1.069\times{10}^{-11} C\cdot N^{-1}$$

$$d_{31}= 10.69 pC\cdot N^{-1}$$

This calculation was repeated for the next 95 maxima of the current signal in the tensile region to determine the average and standard deviation. Then, the calculation was repeated for each strain level, for which the values were reported in the main text. Notably, data from the compressive region was excluded, as the standalone thin-film sample may experience buckling under compression.

1. **Direct Piezoelectric Effect**


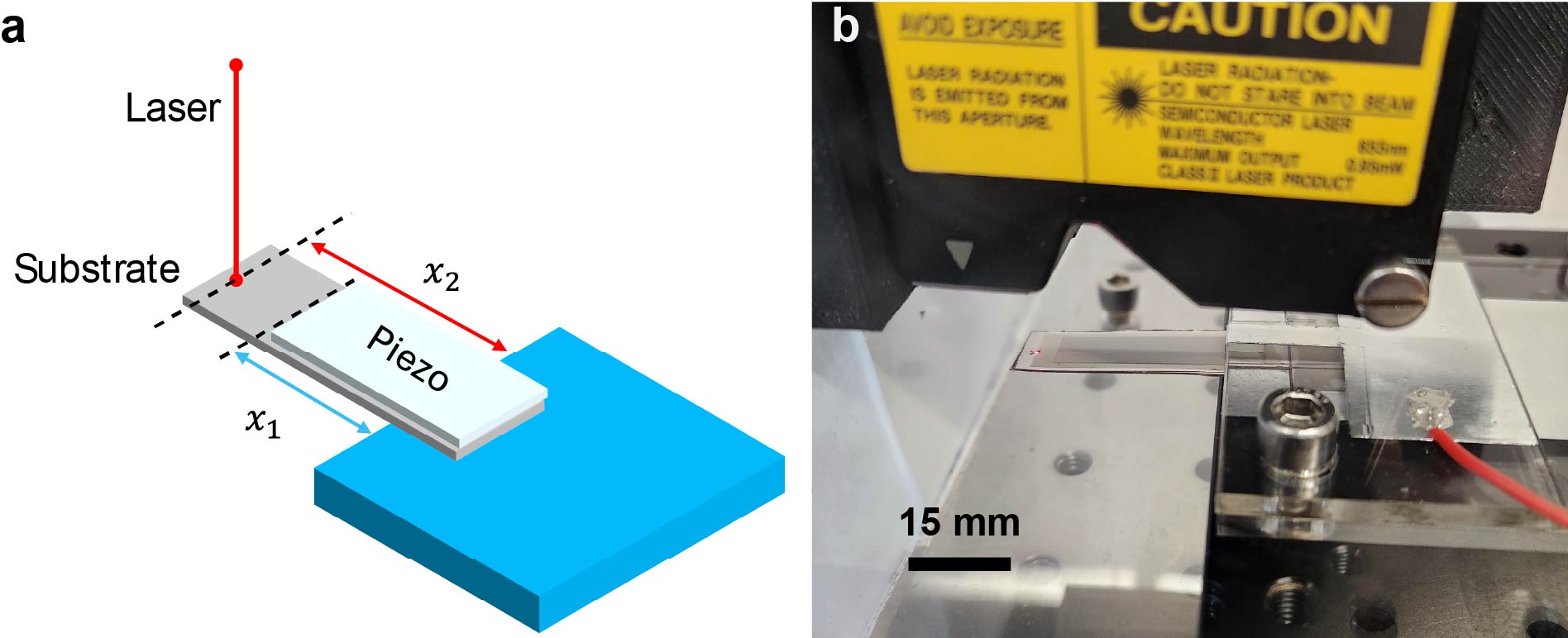


**Figure S9**. a) Schematic of the cantilever-like PVDF-TrFE bender. b) Photograph of the experiment setup.

An estimation of the piezoelectric constant e_31_ for piezoelectric benders attached as cantilever beams was proposed by Mazzalai et al., given by the following **Equation S6**^[7]^:

$$\begin{aligned} e_{31}\approx-\frac{1}{3}\left[ \frac{Y_{b}\left( t_{b} \right)^{2}}{\left( 1-v_{b} \right)\left[ x_{1}\left( 2x_{2}-x_{1} \right) \right]} \right]\left( \frac{w_{b}}{w_{P}} \right)\cdot\frac{{\Delta z}_{x_{2}}}{V_{in}}\#\left( S6 \right) \end{aligned}$$

Where:

- *Y_b_* is the Young’s modulus of the substrate (N m^-2^),
- *t_b_* is the thickness of the substrate (m),
- *v*_b_ is Poisson’s ratio of the substrate,
- *x_1_*, *x_2_* are the distances from the piezoelectric layer to the cantilever edge and from the laser measurement point to the cantilever edge (m), respectively (see Figure S9),
- *w_b_* is the width of the substrate (m),
- *w_p_* is the width of the piezoelectric layer (m),
- Δ*z_x2_* is the piezoelectric-induced displacement at the measurement point (m), and
- *V_in_* is the input/driving voltage (V).

By definition, the piezoelectric constant d_31_ is related to e_31_​ by:

$$d_{31}=\frac{e_{31}}{c_{11}}$$

where *c_11_* is Young’s modulus of the piezoelectric material (Pa or N m^-2^). From here, we denote *c_11_* as *Y_p_*. Substituting this relationship into Equation S6 yields **Equation S7**,

$$\begin{aligned} d_{31}\approx-\frac{1}{3}\left[ \frac{Y_{b}\left( t_{b} \right)^{2}}{Y_{p}\left( 1-v_{b} \right)\left[ x_{1}\left( 2x_{2}-x_{1} \right) \right]} \right]\left( \frac{w_{b}}{w_{P}} \right)\cdot\frac{{\Delta z}_{x_{2}}}{V_{in}} \#\left( S7 \right) \end{aligned}$$

The Young’s modulus of 3D printed PVDF-TrFE (​*Y_p_*) was obtained from the DMA experiment described in the previous section. The RSA-G2 DMA system recorded stress-strain data, with Figure S10a showing the stress-strain curve of the standalone PVDF-TrFE sample subjected to 1.0% cyclic strain at 1 Hz. The sample exhibited hysteretic behavior, a common characteristic of ductile materials under cyclic strain within the elastic deformation limit. As expected, under compression, the thin-film sample experienced buckling, causing saturation in stress readings. However, this buckling was temporary, as a repeatable stress-strain relationship was later observed when the strain returned to tension.


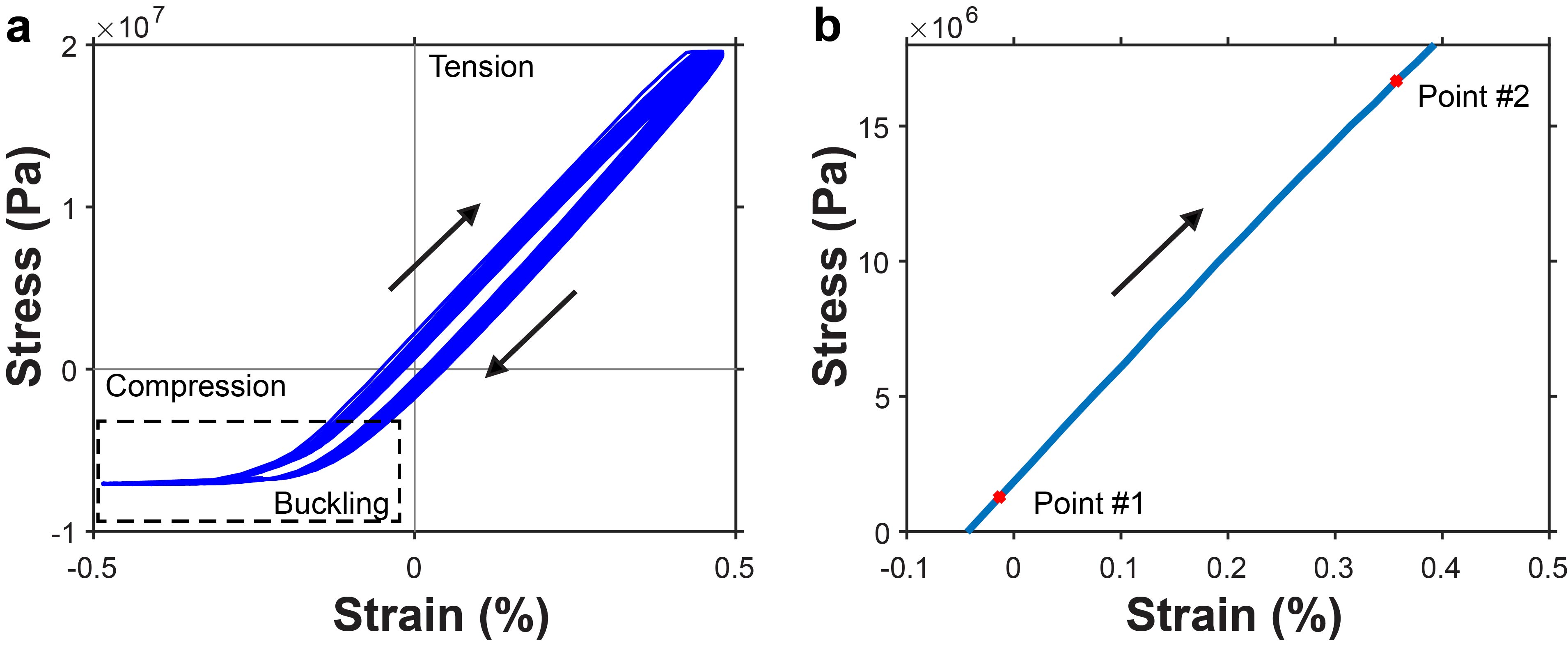


**Figure S10**. a) Stress-strain curve recorded for 1.0%/sec cyclic strain for 100 cycles. b) Zoom-in view for one stress-strain line in the tensile region. Arrows indicate the direction of the applied strain.

Figure S10b presents a zoomed-in view of one stress-strain cycle in the tensile region. From two selected data points:

- Point #1: ε_1_ = - 0.0135 %, σ_1_ = 1.273 MPa
- Point #2: ε_2_ = 0.3572 %, σ_2_ = 16.667 MPa

We calculate the Young’s modulus of 3D printed PVDF-TrFE as:

$$Y_{p}=\frac{\sigma_{2}-\sigma_{1}}{\varepsilon_{2}-\varepsilon_{1}}=\frac{\left( 16.667-1.273 \right)\times{10}^{6} Pa}{\left[ 0.3572-\left( -0.0135 \right) \right]\times0.01}=4.153 GPa$$

This calculated *Y_p_* value is consistent with the supplier’s specification (> 3 GPa).^[8]^

It should be noted that this Young’s modulus shows a discrepancy with values reported by other studies,^[9,10]^ which typically range in the hundreds of MPa. This discrepancy arises from differences in fabrication methods, as electrospun PVDF-TrFE tends to have a lower modulus due to its porous structure, whereas DIW printing preserves the original mechanical strength of the material, similar to the spin-coating method.^[11]^

We then applied this value of Young’s modulus to Equation S7, with:

- *Y_b_* = 193 GPa, *v*_b_ = 0.3, *t_b_* = 0.1 mm, *w_b_* = 12 mm
- *Y_p_* = 4.15 GPa, *w_p_* = 8 mm, *x_1_* = 25 mm, *x_2_* = 28 mm.

This yields:

$$d_{31}\approx\left( -4.2863\cdot{10}^{-4} \right)\cdot\frac{{\Delta z}_{x_{2}}}{\Delta V_{in}}$$

We apply this formula to experimental data from Figure 3C in the main text. Under a driving voltage of ±250 V (sinusoidal at 1 Hz), the four PVDF-TrFE benders produced peak-to-peak actuation displacements of 12.657 µm, 10.395 µm, 12.324 µm, and 13.689 µm, respectively.

E.g., for the first bender,

$$d_{31}\approx\left( -4.2863\cdot{10}^{-4} \right)\cdot\frac{12.657\cdot10^{-6}m}{500 V}$$

$$d_{31}=-1.0850\cdot{10}^{-11} m\cdot V^{-1}$$

$$d_{31}=-10.850 pm\cdot V^{-1}$$

We observe that the *d_31_* ​value computed from the direct piezoelectric response (10.69 pC·N^-1^) is in close agreement with the d_31_ ​value estimated from the inverse piezoelectric response (10.85 pm·V^-1^), demonstrating the consistency and reliability of our measurement methods.

It is important to note that the d_31_ piezoelectric constant can be expressed in two equivalent units: C·N^-1^ or m·V^-1^.

**Signal-to-Noise Ratio (SNR) and Minimum Detectable Force Analysis**

The piezoelectric voltage signals obtained from the unpoled and poled 3D printed PVDF-TrFE sensors (Figure 4B) were analyzed to evaluate their signal-to-noise ratios (SNRs).

1. **Raw Data and Data Filtering**


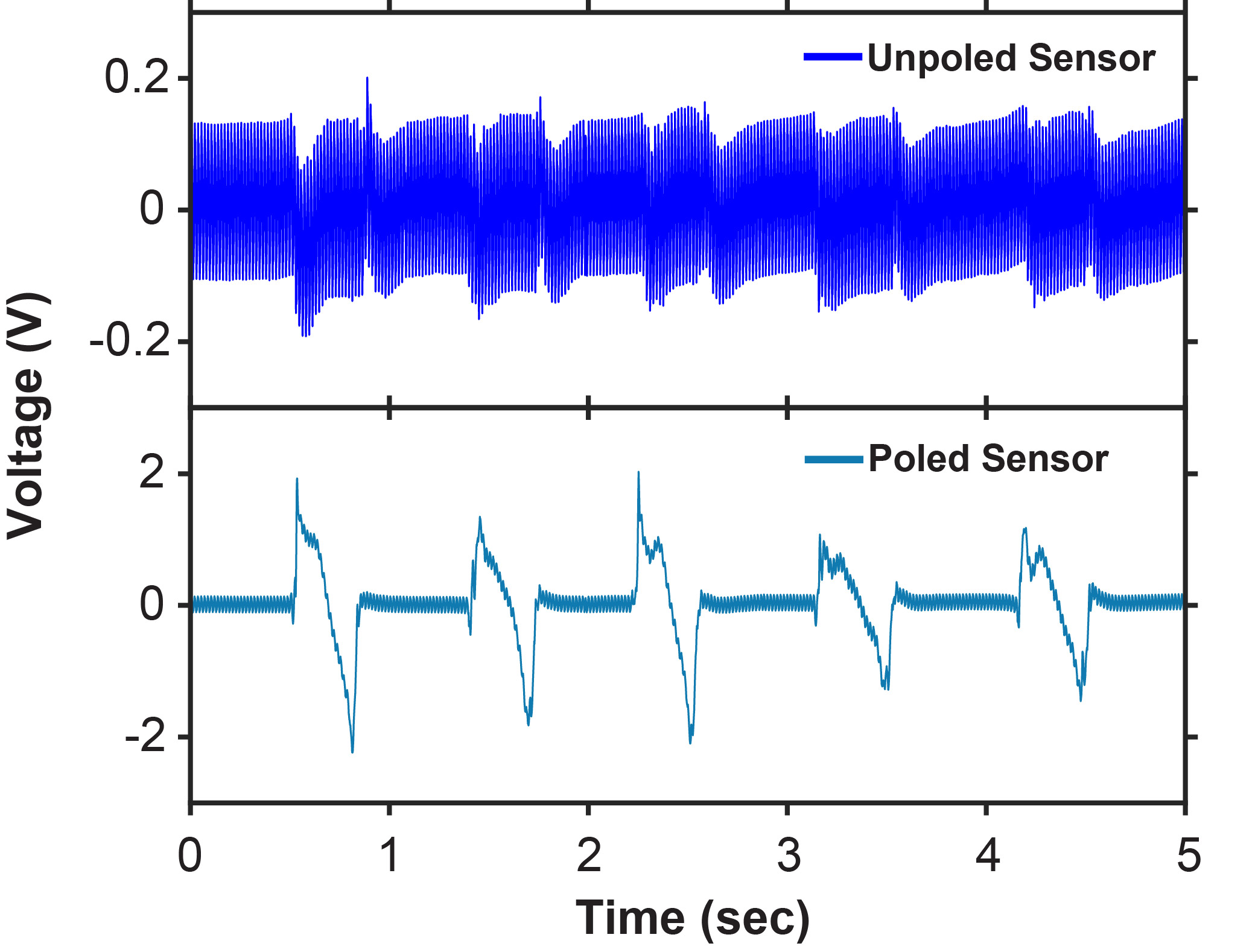


**Figure S11**. Raw voltage outputs collected from unpoled and poled 3D printed PVDF-TrFE sensors.

As shown in Figure S11, the raw voltage outputs from both sensors contained substantial electrical noise superimposed on the piezoelectric response. To extract the useful sensor signal, a digital low-pass filter with a cutoff frequency of 60 Hz was applied to the raw data using a MATLAB script. This filtering step produced smoothed waveforms that represented the primary piezoelectric response while suppressing high-frequency noise components (Figure S12). In practical applications, this low-pass filtering could be implemented using a simple passive RC filter.


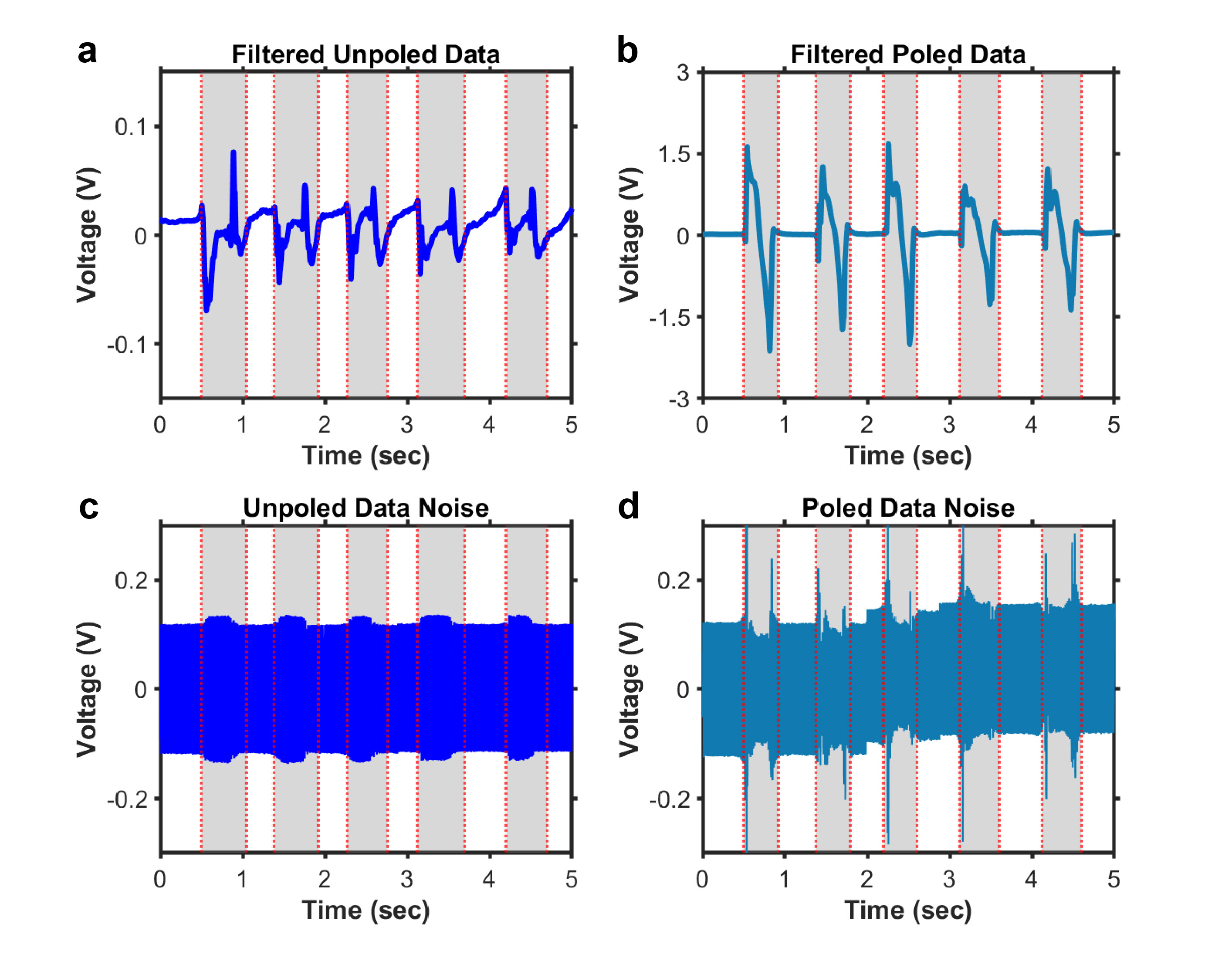


**Figure S12**. Components of voltage data collected from 3D printed PVDF-TrFE sensors after applying a low-pass filter with a cutoff frequency of 60 Hz. a) Filtered voltage (useful) signal from the unpoled sensor, b) filtered voltage (useful) signal from the poled sensor, c) noise signal from the unpoled sensor, and d) noise signal from the poled sensor. The gray regions bounded by red dashed lines indicate the portion of data used for SNR calculations.

Noise was then calculated as the difference between the raw voltage and the low-pass-filtered (useful) signal, i.e.

$$V_{noise}\left( t \right)=V_{raw}\left( t \right)-V_{signal}\left( t \right)$$

This approach ensured that both the useful signal and the noise were characterized under identical operating and loading conditions. It is worth noting that despite the difference in the magnitudes of their piezoelectric responses, both sensors exhibited similar noise levels (ca. ±100 mV).

1. **Root-Mean-Square (RMS) Calculation**

For SNR evaluation, only the portions of the signals corresponding to active sensing periods were considered, where mechanical force was applied (Figure S12; gray regions bounded by red dashed lines). Within these regions, the noise floor (*V_noise, RMS_*) for each sensor was calculated from the noise trace using the root-mean-square (RMS) expression given in Equation S8.

$$\begin{aligned} V_{noise, RMS}=\sqrt{\frac{1}{N}\sum_{i=1}^{N} V_{noise, i}^{2}}\#\left( S8 \right) \end{aligned}$$

The resulting noise RMS values were::

- *V_noise, RMS_* (unpoled) = 79.4 mV
- *V_noise, RMS_* (poled) = 92.4 mV.

Similarly, the RMS output voltage during tapping (*V_signal, RMS_*) of each sensor was calculated from the low-pass-filtered (useful) signal using Equation S9:

$$\begin{aligned} V_{signal,RMS}=\sqrt{\frac{1}{N}\sum_{i=1}^{N} V_{signal,i}^{2}}\#\left( S9 \right) \end{aligned}$$

The results were:

- *V_signal, RMS_* (unpoled) = 19.5 mV
- *V_signal, RMS_* (poled) = 748.3 mV.

1. **SNR Calculation**

The SNR of each sensor was then calculated using Equation S10:

$$\begin{aligned} {SNR}_{dB}=20\log_{10} \left[ \left( \frac{V_{signal, RMS}}{V_{noise, RMS}} \right) \right]\#\left( S10 \right) \end{aligned}$$

The resulting SNR values were:

- *SNR_dB_* (unpoled) = -12.21 dB
- *SNR_dB_* (poled) = 18.17 dB

The negative SNR value of the unpoled sensor indicated that its piezoelectric signal was weaker than the background noise and could be easily masked. Conversely, after electrical poling, the poled sensor exhibited a positive and substantially higher SNR value.

1. **Minimum Detectable Force**

The 3D printed piezoelectric sensors were dynamic sensors. Therefore, the frequency (or rate of change) of the input force directly affected the magnitude of minimum detectable force.

For this analysis, the 0.2%/sec strain rate DMA dataset was used because the DMA equipment provided reliable force measurements. The 0.6%/sec and 1.0%/sec strain rate DMA datasets were not used, as buckling occurred during the compressive phase.


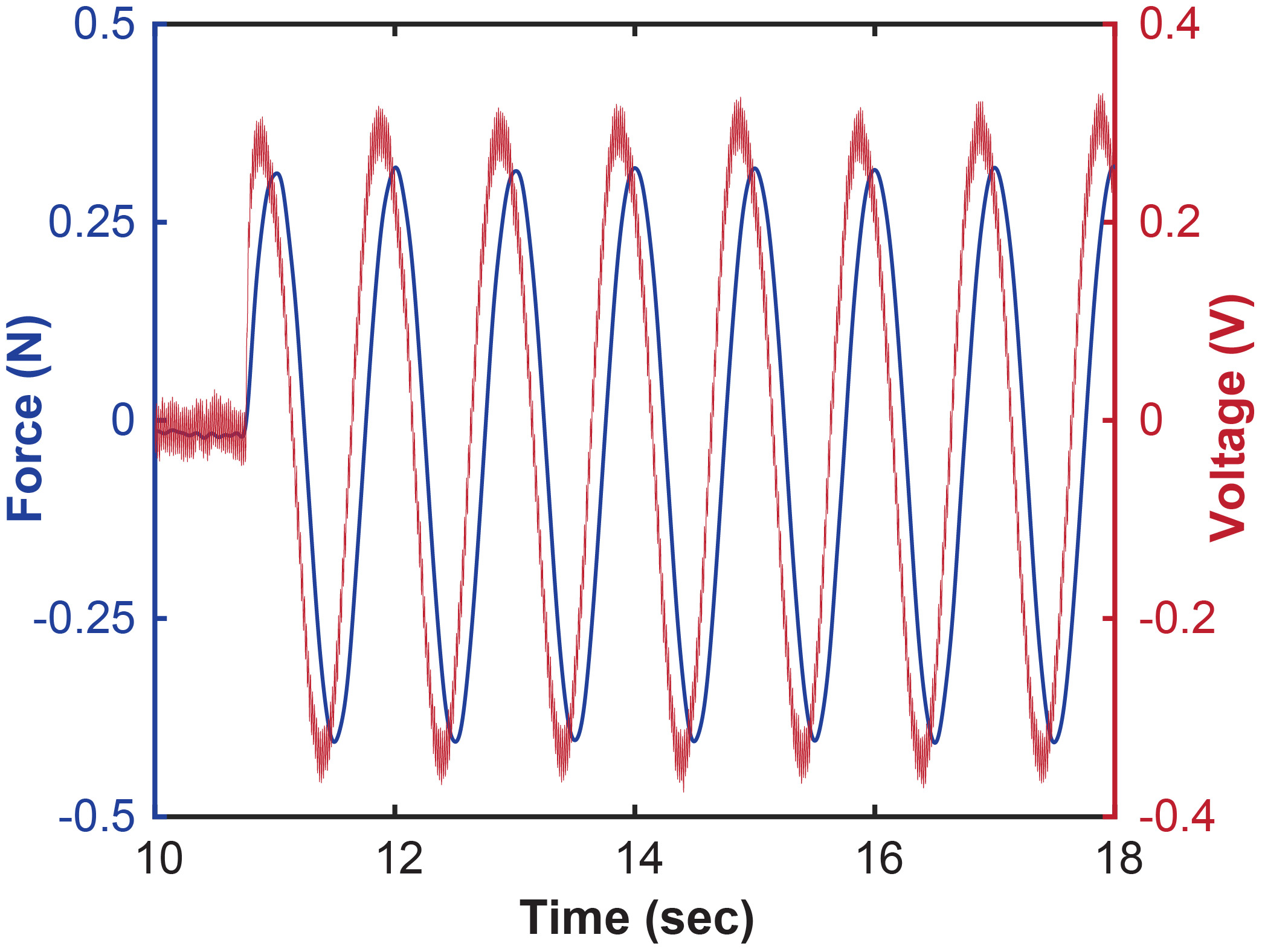


**Figure S13**. A portion of input force and raw voltage output collected from the 0.2%/sec strain rate DMA experiment.

Figure S13 showed a portion of the input force profile together with the corresponding raw voltage signal. A low-pass filter was applied to the raw voltage signal to obtain the useful piezoelectric response, and the noise component was again calculated as the difference between the raw and filtered signals, both shown in Figure S14.


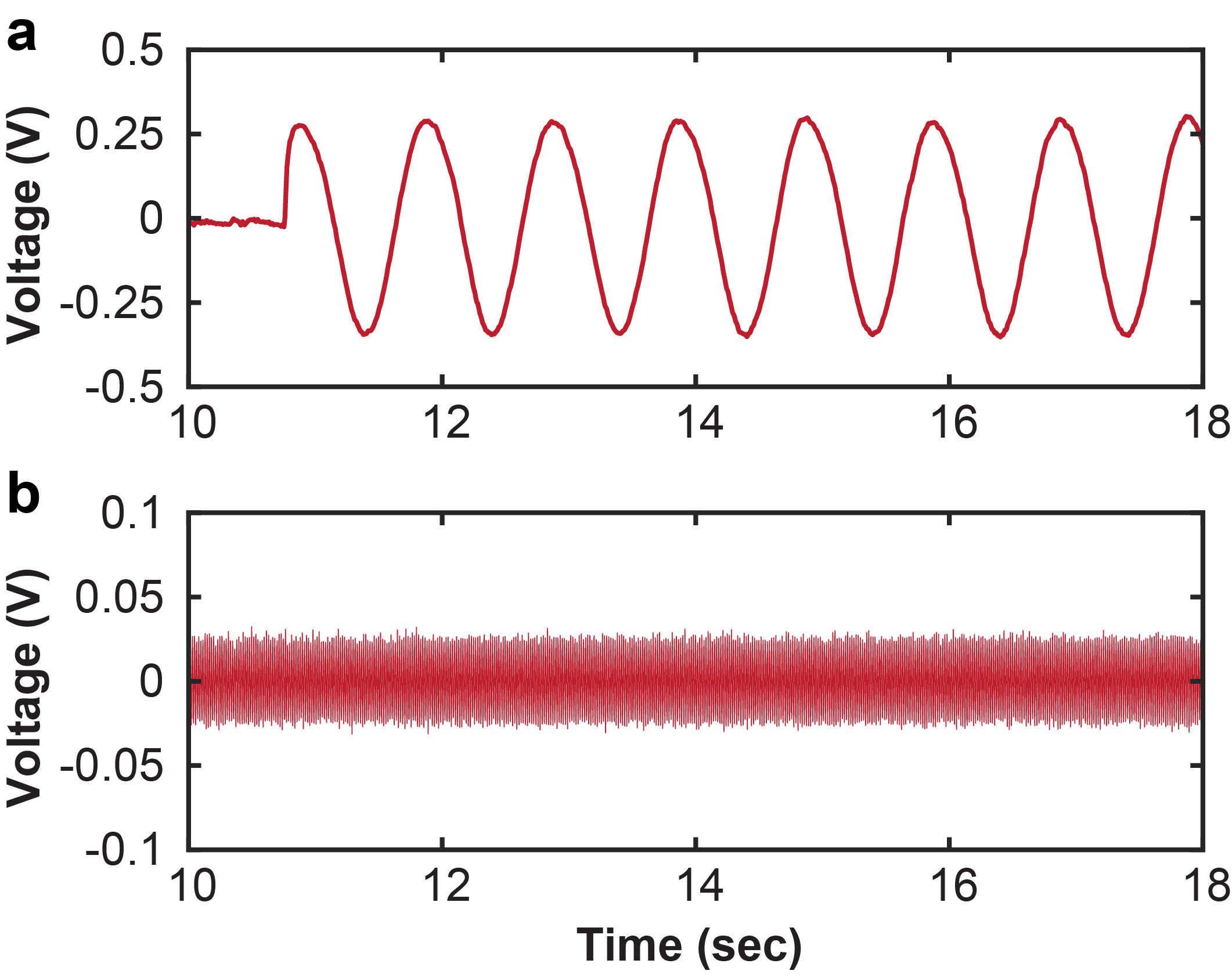


**Figure S14**. Components of voltage data collected from the 3D printed PVDF-TrFE DMA sample after applying a low-pass filter with a cutoff frequency of 60 Hz. a) Filtered voltage (useful) signal, and b) noise signal.

A MATLAB script was used to calculate the average peak-to-peak force from the DMA data, as well as the corresponding average peak-to-peak filtered voltage from the sensor.

$$\bar{F}_{p2p}=0.721 N$$

$$\bar{V}_{p2p}=0.635 V$$

(Under the 0.2%/sec loading condition, the 3D printed piezoelectric sensors experienced two peak-to-peak cycles per second (1 Hz), hence the effective force application rate was $\dot{F}=2\cdot\bar{F}_{p2p}/ 1 sec =1.442 N/s$.)

The force sensitivity (S_F_) of the 3D printed sensor was calculated as follows.

$$S_{F}=\frac{\bar{V}_{p2p}}{\bar{F}_{p2p}}=\frac{0.635 V}{0.721 N}=0.882 V/N$$

The average peak value of the background noise in the collected sensor data was calculated as well, $\bar{V}_{noise, peak}=0.039 V$.

Finally, the minimum detectable force, *F_MD_*, masked by the background noise was calculated as:

$$F_{MD}=\frac{\bar{V}_{noise}}{S_{F}}=\frac{0.039 V}{0.882 V/N}=0.044 N$$

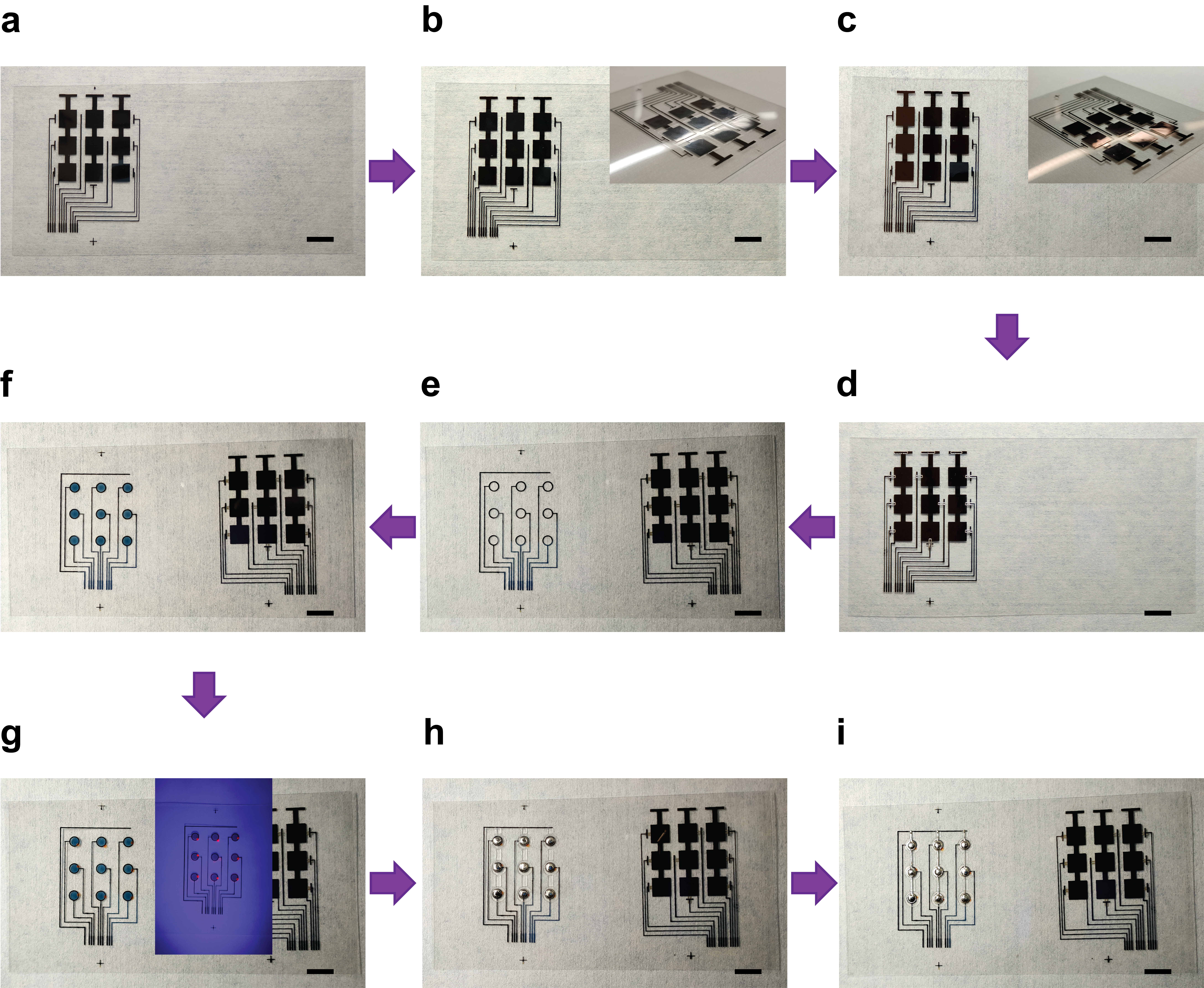


**Figure S15**. Major steps in the printing process of multifunctional flexible devices. 3D printing of a) AgNPs to form the bottom electrodes and interconnects for top electrodes, b) PVDF-TrFE, inset shows the perspective view, c) AgNPs to form the top electrodes, inset shows the perspective view, d) silver epoxy to connect the top electrodes to the corresponding interconnects, e) AgNPs to form the interconnects for the QLEDs, f) PEDOT:PSS, g) TFB and QDs to form the active layers, inset shows the photoluminescence of QDs under near-UV light, h) silicone and EGaIn to form the insulating layer and cathodes, i) silver epoxy to connect all of the cathodes to a common ground interconnect. (Scale bars = 12 mm)

The two sides of a PET film were labelled as Face A and Face B. Steps (a) – (d) were printed on Face A, whereas steps (e) – (i) were printed on Face B. After the fabrication of the multifunctional device, polydimethylsiloxane (PDMS) was poured onto the QLEDs to form flexible, transparent encapsulation. Lastly, two 15-pin flat flexible cables (FFCs) were attached to the interconnects of the PVDF-TrFE sensor and QLED arrays for data transmission and power supply, respectively.


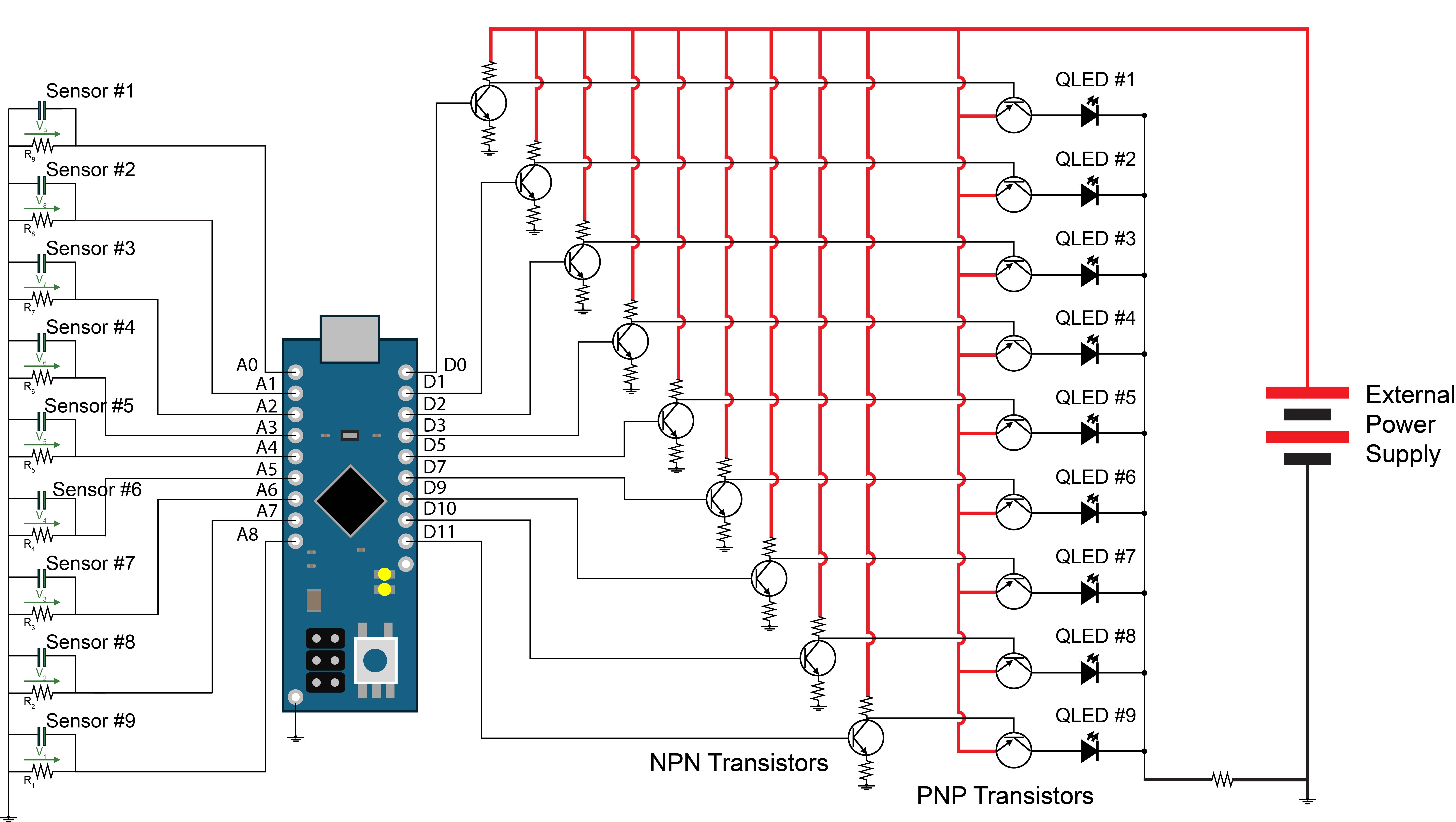


**Figure S16**. Circuit diagram of the 3D printed multifunction hybrid device with the piezoelectric sensor and QLED arrays.

For simpler visual presentation, the piezoelectric sensors are simply modelled as capacitors, neglecting the current-generating components.

The microcontroller is an Arduino Micro, the NPN transistors are 2N3904, and the PNP transistors are 2N3906. Resistors *R_1_* to *R_9_* (connected in parallel across the piezoelectric sensors) have a resistance of 100 MΩ, whereas the remaining, unlabelled resistors in the diagram have a resistance of 1 kΩ.


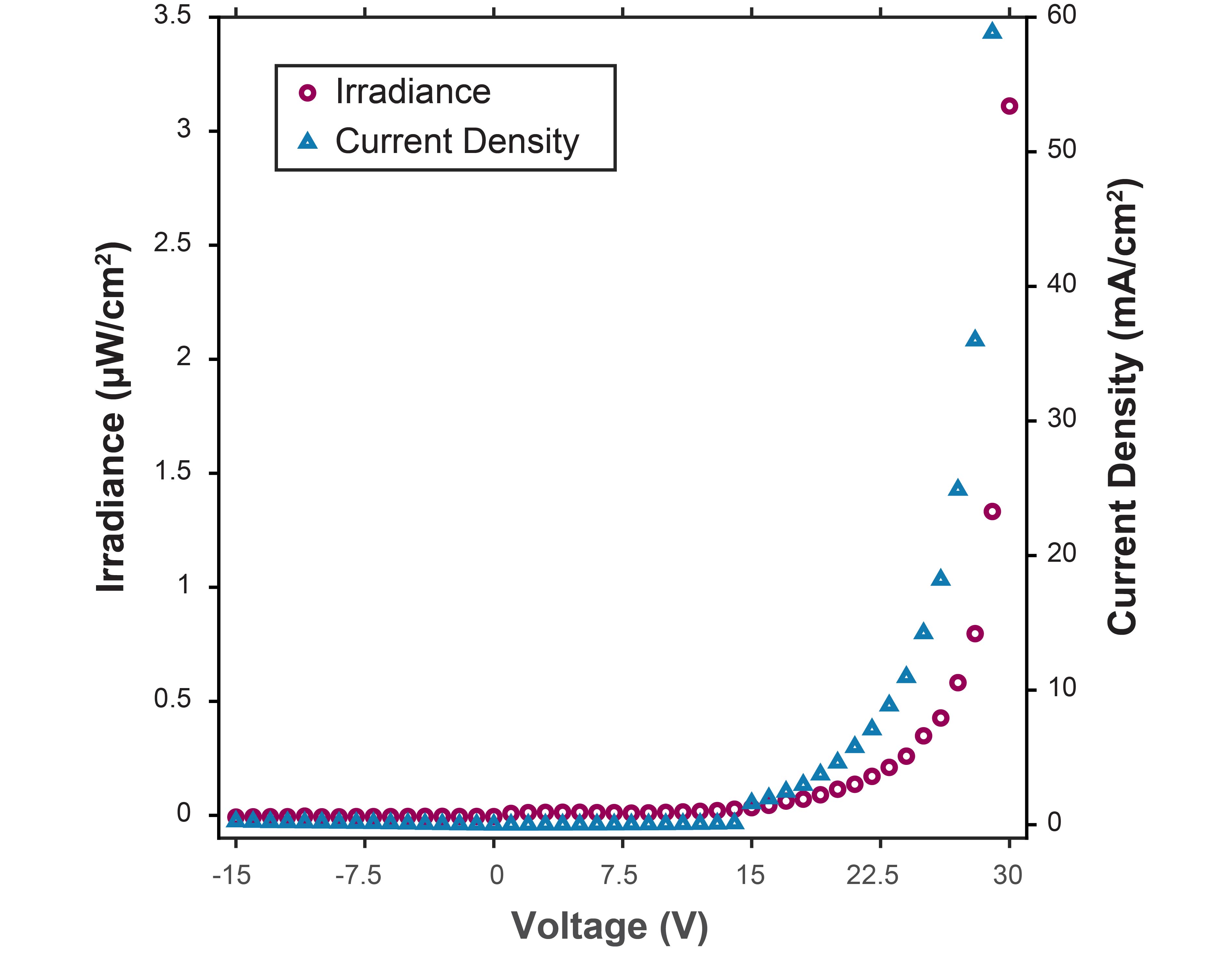


**Figure S17**. Current-voltage (I-V) and irradiance-voltage plots of a 3D printed QLED under positive and negative voltages.

**Table S1**. Printing parameters used for fabricating multifunctional hybrid electronics.

| **Material** | **Nozzle Type** | **Nozzle Inner Diameter**  **(µm)** | **Pressure**  **(kPa)** | **Speed**  **(mm/min)** | **Printhead Height**  **(µm)** | **Dispensing Time**  **(sec)** | **Curing Conditions** |
| --- | --- | --- | --- | --- | --- | --- | --- |
| AgNPs | Stainless steel | 80 | 0.1 | 600 | 20 | - | 80 °C, 15 mins |
| PVDF-TrFE | Stainless steel | 250 | 45 | 200 | 50 | - | 80 °C, 10 mins |
| Ag Paint | Stainless steel | 250 | 120 | 500 | 100 | - | 80 °C, 30 mins |
| PEDOT:PSS | Stainless steel | 100 | 5 | 450 | 50 | - | 80 °C, 10 mins |
| TFB | Stainless steel | 100 | 0.1 | 450 | 10 | - | Room temperature |
| QDs | Stainless steel | 100 | 0.1 | 450 | 10 | - | Room temperature |
| Silicone | Tapered polypropylene | 100 | 420 | 600 | 100 | - | Room temperature |
| EGaIn | Tapered polypropylene | 100 | 35 | - | 1.5 (cm) | 0.10 | - |
| Ag Epoxy | Tapered polypropylene | 250 | 100 - 150 | 100 | 200 | - | 80 °C, 5 mins |
| PDMS | - | - | - | - | - | - | 80 °C, 30 mins |

**References**

[1] H. Zhu, T. Miyashita, M. Mitsuishi, “Energy Storage Behaviors in Ferroelectric Capacitors Fabricated with Sub-50 Nm Poly(Vinylidene Fluoride) Langmuir–Blodgett Nanofilms.” *Polymer Journal* 51, 8 (2019): 795–801. https://doi.org/10.1038/s41428-019-0194-3.

[2] Y. L. Kong, I. A. Tamargo, H. Kim, et al., “3D Printed Quantum Dot Light-Emitting Diodes.” *Nano Letters* 14, 12 (2014): 7017–23. https://doi.org/10.1021/nl5033292.

[3] J. Kim, H. J. Shim, J. Yang, et al., “Ultrathin Quantum Dot Display Integrated with Wearable Electronics.” *Advanced Materials* 29, 38 (2017): 1700217. https://doi.org/10.1002/adma.201700217.

[4] A. Dive, J. Varley, S. Banerjee, “In 2 O 3 − Ga 2 O 3 Alloys as Potential Buffer Layers in Cd Te Thin-Film Solar Cells.” *Physical Review Applied* 15, 3 (2021): 034028. https://doi.org/10.1103/PhysRevApplied.15.034028.

[5] J. Li, N. Wu, “Semiconductor-Based Photocatalysts and Photoelectrochemical Cells for Solar Fuel Generation: A Review.” *Catalysis Science & Technology* 5, 3 (2015): 1360–84. https://doi.org/10.1039/C4CY00974F.

[6] *IEEE* *Standard on Piezoelectricity*, ANSI/IEEE Std 176-1987, (1988).

[7] A. Mazzalai, D. Balma, N. Chidambaram, Li Jin, P. Muralt, “Simultaneous Piezoelectric and Ferroelectric Characterization of Thin Films for MEMS Actuators.” *2013 Joint IEEE International Symposium on Applications of Ferroelectric and Workshop on Piezoresponse Force Microscopy (ISAF/PFM)*, July, 363–66 (2013). https://doi.org/10.1109/ISAF.2013.6748724.

[8] “Piezo PVDF-TrFE Film - Page 1 - Piezoelectric PVDF & PVDF-TrFE,” can be found under https://piezopvdf.com/piezo-pvdf-trfe-film/, **2025**.

[9] A. Poudel, M. A. Fernandez, S. A. M. Tofail, M. J. P. Biggs, “Boron Nitride Nanotube Addition Enhances the Crystallinity and Cytocompatibility of PVDF-TrFE.” *Frontiers in Chemistry* 7. May (2019): 364. https://doi.org/10.3389/fchem.2019.00364.

[10] A. Wang, M. Hu, L. Zhou, X. Qiang, “Self-Powered Well-Aligned P(VDF-TrFE) Piezoelectric Nanofiber Nanogenerator for Modulating an Exact Electrical Stimulation and Enhancing the Proliferation of Preosteoblasts.” *Nanomaterials* 9, 3 (2019): 349. https://doi.org/10.3390/nano9030349.

[11] D. G. Jeong, H. H. Singh, M. S. Kim, J. H. Jung, “Effect of Centrifugal Force on Power Output of a Spin-Coated Poly(Vinylidene Fluoride-Trifluoroethylene)-Based Piezoelectric Nanogenerator.” *Energies* 16, 4 (2023): 1892. https://doi.org/10.3390/en16041892.

**Movie Captions**

Movie S1. Interactive gaming with 3D printed piezoelectric sensors.

Movie S2. Tactile sensing “electronic skin” with 3D printed piezoelectric sensors.

Movie S3. 3D printing of the piezoelectric sensor array of a multifunctional hybrid device.

Movie S4. 3D printing of the quantum dot LED array of a multifunctional hybrid device.

Movie S5. 3D printed multifunctional hybrid device with integrated piezoelectric sensor and quantum dot LED arrays.
